# Modulation of Human Leukocyte Antigen-C by Human Cytomegalovirus Stimulates KIR2DS1 Recognition by Natural Killer Cells

**DOI:** 10.3389/fimmu.2017.00298

**Published:** 2017-03-29

**Authors:** Kattria van der Ploeg, Chiwen Chang, Martin A. Ivarsson, Ashley Moffett, Mark R. Wills, John Trowsdale

**Affiliations:** ^1^Department of Pathology, University of Cambridge, Cambridge, UK; ^2^Department of Medicine, University of Cambridge, Cambridge, UK

**Keywords:** natural killer cells, human cytomegalovirus, killer Ig-like receptor, KIR2DS1, HLA-C

## Abstract

The interaction of inhibitory killer cell Ig-like receptors (KIRs) with human leukocyte antigen (HLA) class I molecules has been characterized in detail. By contrast, activating members of the KIR family, although closely related to inhibitory KIRs, appear to interact weakly, if at all, with HLA class I. KIR2DS1 is the best studied activating KIR and it interacts with C2 group HLA-C (C2-HLA-C) in some assays, but not as strongly as KIR2DL1. We used a mouse 2B4 cell reporter system, which carries NFAT-green fluorescent protein with KIR2DS1 and a modified DAP12 adaptor protein. KIR2DS1 reporter cells were not activated upon coculture with 721.221 cells transfected with different HLA-C molecules, or with interferon-γ stimulated primary dermal fibroblasts. However, KIR2DS1 reporter cells and KIR2DS1^+^ primary natural killer (NK) cells were activated by C2-HLA-C homozygous human fetal foreskin fibroblasts (HFFFs) but only after infection with specific clones of a clinical strain of human cytomegalovirus (HCMV). Active viral gene expression was required for activation of both cell types. Primary NKG2A^−^KIR2DS1^+^ NK cell subsets degranulated after coculture with HCMV-infected HFFFs. The W6/32 antibody to HLA class I blocked the KIR2DS1 reporter cell interaction with its ligand on HCMV-infected HFFFs but did not block interaction with KIR2DL1. This implies a differential recognition of HLA-C by KIR2DL1 and KIR2DS1. The data suggest that modulation of HLA-C by HCMV is required for a potent KIR2DS1-mediated NK cell activation.

## Introduction

Since the discovery of natural killer (NK) cells more than 40 years ago (1–3), the interaction of inhibitory NK cell receptors with human leukocyte antigen (HLA) class I molecules has been characterized in detail. This led to new insights into NK cell differentiation, education, and function. However, ligands of most activating receptors, including activating killer cell Ig-like receptors (KIRs), are yet to be discovered.

*KIR* genes are members of the immunoglobulin (Ig) superfamily, encoded in the leukocyte receptor complex (LRC) on chromosome 19q14.3 (4). KIR molecules express either two or three extracellular Ig-like domains (2D or 3D) and consist of either a long (designated “L”) or short (designated “S”) cytoplasmic domain. KIRs with long cytoplasmic domains are inhibitory (iKIRs) and contain ITIMs. Activating KIRs (aKIRs) have a short cytoplasmic tail and transmit activating signals through the interaction with DAP12, which contains an ITAM (4).

Most iKIRs recognize certain allotypes of HLA class I. In general, allelic products of *KIR2DL1* bind to the C2 group of HLA-C molecules (C2-HLA-C) characterized by Asn77 and Lys80 (5), while KIR2DL2 and -2DL3, which are alleles at the same locus, recognize the C1 group (C1-HLA-C, Ser77, and Asn80) (6–8). These structural motifs were originally thought to be essential for the engagement of KIRs only on HLA-C. However, KIR2DL2 can also bind HLA-B46:01 and -B73:01 alleles, which have C1-related motifs at residues 77–83 (9). Furthermore, KIR2DL2 and -L3 receptors can bind many HLA-C alleles irrespective of -C1 or -C2 group (10, 11).

The extracellular parts of iKIRs and aKIRs are highly homologous and share conserved amino acid sequences, as “paired” receptors (11, 12). The balance between inhibitory and activating signaling through these paired receptors is tightly regulated by NK cells. Dysregulation of this balance might lead to autoimmunity or infectious diseases (13, 14). How the signaling is controlled by NK cells, however, is not completely understood, mainly due to uncertainty over the ligands and functions of aKIRs. The aKIR members seem to have evolved more rapidly than iKIRs, possibly through selection pressure imposed by pathogens (15, 16). If this hypothesis is true, it suggests that aKIR binding may be influenced by pathogen-derived proteins. Notably, KIR2DS1 and -2DS2 counterparts in chimpanzees, respectively, bind C2- and C1-HLA-C with high avidity compared to their inhibitory paired receptors (17). This indicates that the loss of binding by KIR2DS2, or highly reduced binding of KIR2DS1, to HLA-C is a product of human-specific evolution.

Most interactions of aKIRs and HLA class I molecules are very weak or undetectable (17–23). The best studied aKIR is KIR2DS1 and many studies have found that it binds C2-HLA-C (10, 11, 17, 24–35). However, this binding is much weaker compared to KIR2DL1 (10, 25, 27). Using surface plasmon resonance (SPR) analysis, Stewart and colleagues demonstrated that KIR2DS1 tetramer-binding avidity to the soluble HLA-Cw4/beta-2 microglobulin (β_2_M)/peptide complex is approximately four times lower than KIR2DL1: dissociation constants (*K*_d_) of 7.2 and 30 μM, respectively. In addition, amino acid substitution of the peptide at amino acid 7 or 8 drastically reduced KIR2DS1 tetramer binding to the HLA-Cw4 complex, indicating that the binding is peptide dependent (27). Both Moesta et al. and Hilton et al. demonstrated that KIR2DS1-Fc binds a range of C2-HLA-C allotypes linked to microbeads with different avidity (11, 17). Biassoni and colleagues demonstrated that the amino acid at position 70, a threonine in KIR2DL1 and a lysine in KIR2DS1, is the key residue that differentiates binding between KIR2DL1 and -2DS1. Substituting the threonine to a lysine in KIR2DL1-Fc prevented binding to 721.221 transfected with HLA-Cw4 (221-Cw4). When lysine was substituted for a threonine in KIR2DS1-Fc, the binding to 221-Cw4 was restored (25).

The rationale for KIR2DL1 and KIR2DS1 both binding C2-HLA-C would be understandable if the activating receptor was sensitive to structural changes in the HLA molecule, or bound an alternative molecule, induced by viral infection (18, 27, 36–42). For example, in mice, the cytomegalovirus (CMV)-encoded MHC class I homolog m157 is directly recognized by the activating Ly49H receptor (43). The mouse Ly49 receptor family serves a similar role to KIRs in humans, although KIRs and Ly49 receptors are from different molecular families. KIRs play an important role in human cytomegalovirus (HCMV) infections (44–47). For instance, a recent study demonstrated that KIR2DS1^+^ decidual NK (dNK) cells degranulated after engaging with HCMV-infected decidual stromal cells (DSC), suggesting an increased ability of KIR2DS1-expressing dNK cells to respond to placental HCMV infection (47). Della Chiesa et al. have reported that HCMV can drive NK cell maturation in the absence of NKG2C in patients with hematological malignancies. These patients received umbilical cord blood transplantation from NKG2C^−/−^ donors and when HCMV reactivation occurred, an expansion of NKG2A^−^ NK cells expressing aKIRs was measured, particularly KIR2DS1 and KIR3DS1 (45). This finding is consistent with KIR2DS1 recognizing a ligand on HCMV-infected cells.

To probe the potential influence of HCMV on KIR2DS1 recognition, we designed a mouse 2B4 T cell hybridoma carrying an NFAT-green fluorescent protein (GFP) reporter. Our results suggest that modulation of HLA-C by HCMV is required for a potent KIR2DS1-mediated NK cell activation.

## Materials and Methods

### Cell Lines and Cell Culture

Reporter cells, 721.221 cells, K562 cells, and primary NK cells were cultured in RPMI-1640 (Sigma-Aldrich, Steinheim, Germany) supplemented with 100 U/ml penicillin, 100 μg/ml streptomycin (Gibco, Paisley, UK), and 10% heat-inactivated Fetal Bovine Serum (Gibco). Human fetal foreskin fibroblasts (HFFFs, Culture Collections—ECACC, UK) and primary dermal fibroblasts (DFs) were cultured in Dulbecco’s Modified Eagle’s Medium (DMEM, Sigma-Aldrich) supplemented with 100 U/ml penicillin, 100 μg/ml streptomycin (Gibco), and 10% heat-inactivated fetal bovine serum. Adherent cells were harvested by washing the cells once with phosphate buffered saline (PBS, Sigma-Aldrich). Then the cells were either detached from the plastic using 0.05% Trypsin/EDTA (Gibco) or Accutase™ (Biolegend, San Diego, CA, USA) for 5 min at 37°C. All cellular experiments were performed at 37°C in 5% CO_2_.

721.221 cells transfected with -A23:01, -B58:01 (Bw4), -B35:01 (Bw6), -C01:02 (C1), -C02:02 (C2), -C03:02 (C1), -C04:01 (C2), -C06:02 (C2), -C07:01 (C1), and -HLA-G were generated in house. 721.221-HLA-A11:02 was provided by Parham (22) (Stanford University, Palo Alto, CA, USA).

### Establishment of the Reporter Cells

The 2B4 T cell hybridoma containing an NFAT-GFP reporter gene (2B4 reporter cells) was kindly provided by Lewis Lanier (43) (University of California San Francisco, USA). KIR2DL1*003, -2DL2*001, -2DS1*002, and -2DS1^(K70T)^ reporter cells were generated as follows. First, pMX-neo constructs containing cDNA from a chimeric adaptor protein recombinant was used to transduce the 2B4 reporter cells. The chimeric adaptor consists of DAP12 and a cytoplasmic tail of DAP10 with spacer sequences in between. Then cDNA of the indicated KIRs was subcloned into a pMX-puro construct. For constructing 2DL1–2DS1TM (KIR2DL1 reporter) and 2DL2–2DS1TM (KIR2DL2 reporter) chimeric molecules, 5′-CCTGCACGTTCTGATTGGGACCTCAGT-3′ and 5′-CCCAATCAGAACGTGCAGGTGTCGGGGGTT-3′ primers were used. 5′-AGTCGCATGACGCAAGACCTGGCAGGG-3′ and 5′-GGTCTTGCGTCATGCGACTGATGGAG-3′ primers were used for constructing the KIR2DS1^(K70T)^ reporter cell. Retroviruses were packaged in Phoenix-eco cells (generously provided by Lewis Lanier) using the non-modified polyethyleneimine (PEI, Sigma-Aldrich) reagent as described by Ehrhardt et al. (48). After 48 h, supernatant-containing retroviral particles was used to transduce the 2B4 reporter cells by adding Polybrene (8 ng/ml, Sigma-Aldrich) and by spin-infecting the cells at 2,500 rpm (AccuSpin 3R centrifuge, Fisher Scientific, Waltham, MA, USA) for 2 h. Cells expressing the KIRs were purified by surface staining using the PAN2D antibody (clone NKVFS1, Bio-Rad, Hercules, CA, USA), followed by single-cell sorting using the FACS sorter (BD Bioscience, Oxford, UK). The transduction success and the function of the reporter cells were analyzed by immunofluorescent staining and antibody crosslinking, as described below. The LILRB1 reporter cell was provided by Des Jones (Department of Pathology, University of Cambridge) and was constructed as described in Ref. (49).

### Primary Cells

Primary NK cells and DFs were obtained from healthy individuals. Ethical approval for the use of these tissues was given by Addenbrookes National Health Service Hospital Trust institutional review board (Cambridge Research Ethics Committee) and informed written consent was obtained from all volunteers in accordance with the Declaration of Helsinki (LREC 97/092). The primary fibroblasts were fully HLA typed. HFFFs express HLA-A11:01, -A24:02, -B35:02 (Bw6), -B40:02 (Bw6), -C02:02 (C2), and -C04:01 (C2). Donor CMV307 expresses HLA-A01:01, -A26:01, -B08:01 (Bw6), -B27:05 (Bw4), -C07:01 (C1), and -C01:02 (C1). Donor CMV0005 expresses HLA-A02, -A03, -B07 (Bw6), -B13 (Bw4), -C07 (C1), and -C06 (C2). Primary NK cell donors both express KIR2DL1, -2DS1, 2DL3, NKG2A, and were C1/C1-HLA-C. In addition, donor 016 expresses NKG2C and donor 111 expresses KIR3DL1.

For functional NK cell studies, peripheral blood mononuclear cells (PBMCs) were extracted from 30 to 40 ml blood of donor 016 and 111 on a Ficoll-Hypaque density gradient (Lympoprep, Axis-Shield, Dundee, Scotland). The PBMCs were removed from the interface of the plasma and Lymphoprep layers and washed three times with PBS before further use. NK cells were separated from the PBMCs by negative selection using the EasySep™ Human NK cell Enrichment Kit from Stemcell Technologies (Vancouver, BC, Canada).

Dermal biopsies were taken from healthy individuals by Andrew Carmichael (Department of Medicine, University of Cambridge). They were sectioned with a scalpel and were grown beneath cover slips in a six-well culture plate containing Eagle’s Minimum Essential Media (EMEM, GE Healthcare, Little Chalfont, UK) supplemented with 100 U/ml penicillin, 100 μg/ml streptomycin (Gibco), and 10% heat-inactivated fetal calf serum (Life Technologies, Carlsbad, CA, USA). The cells were grown until sufficient cell number was reached and were stored in liquid nitrogen at low passage numbers.

### Coculture Experiments

Human fetal foreskin fibroblasts (10 × 10^3^ cells/well) and DFs (10 × 10^3^ cells/well) were seeded in 96-well flat-bottom culture plates with or without interferon (IFN)-γ (500 U/ml, PeproTech, Rocky Hill, CT, USA) for 72 h or infected with HCMV as described below. After appropriate stimulation/infection time was reached, reporter cells were added to the adherent cells at a concentration of 2 × 10^4^ cells per well, and reporter cells were added directly. Cocultures using K562 cells and 721.221 cells were performed at an E:T ratio of 1:1 and 1:3, respectively. After an overnight coculture, the reporter cells were harvested, and GFP expression was analyzed by flow cytometry.

### Antibody Cross-linking

For antibody crosslinking experiments, anti-mouse IgG-coated microplates (R&D systems, Minneapolis, MN, USA) were used. The plates were incubated for 30 min with 0.2–1 μg per well of PAN2D (clone NKVFS1) or anti-HA (clone HA-7, Sigma-Aldrich) antibodies in PBS at room temperature. After two PBS washes, the reporter cells were added.

### Antibody-Blocking Assays

Unconjugated W6/32 (LEAF™ purified BioLegend), 6A4 (gift from Daniela Pende, Azienda Ospedaliera Universitaria San Martino di Genova), B1.23.2 (eBiosciences, San Diego, CA, USA), IgG2a (LEAF™ purified BioLegend), IgG2b (LEAF™ purified BioLegend), and IgG1 (LEAF™ purified BioLegend) isotype antibodies were added to pre-treated HFFFs. After an incubation of 10–15 min at room temperature and saturating concentration of 1–4 μg per well, the reporter cells were added.

### Flow Cytometry

#### Immunofluorescence Cell-Surface Staining

The cells were harvested and rested at 37°C in 5% CO_2_ for at least 30 min for the recovery of HLA class I molecules on the cell surface. Cells expressing Fc receptors or HCMV-encoded Fc receptors were first blocked with 40% human serum (Sigma-Aldrich) in PBS for at least 5 min before staining. Fractions of 721.221 cells were collected and directly incubated with the appropriate antibody. Cell-surface expression of different receptors was analyzed by immunofluorescent staining using unconjugated monoclonal primary antibodies listed in Table 1, unconjugated anti-HLA-E (3D12, IgG1, BioLegend), biotinylated anti-HLA-A11 (Abcam, IgM, Cambridge, UK), APC-conjugated anti-HLA-Bw6 (IgG1, Miltenyi Biotec, Bergisch Gladbach, Germany), and the appropriate isotype control antibodies. The primary antibodies were added at a concentration of 0.5–1 μg per well in 5% FCS in PBS or 40% human serum in PBS and incubated on ice for 45 min. Following washing, the cells were incubated with secondary antibodies (0.4–1 μg per well), AlexaFluor^®^ 647 (AF647)-conjugated Streptavidin (Life Technologies) and polyclonal anti-mouse IgG conjugated to FITC (BD Pharmingen, San Diego, CA, USA), AF647 (Life Technologies), or R-PE (Thermo Scientific), in 5% FCS with PBS or in 40% human serum with PBS for 30 min on ice. For double staining experiments, the W6/32 antibody or IgG2a isotype control conjugated to AF647 or FITC (BioLegend) was added to the cells for 30 min on ice. The cells were fixed with 1% formaldehyde in PBS before flow cytometry analysis.

**Table 1:** **List of antibodies and their target epitopes where known**.

Antibody	Isotype/*supplier*	Recognition	Specific HLA molecules	Reference
W6/32	IgG2a/*BioLegend, Hybridoma*	β_2_M bound, fully assembled human leukocyte antigen (HLA) class I molecules. The epitope is not known. It includes residues on β_2_M, α2, and α3 domains	HLA-A, -B, -C and HLA-E, -G	(50, 51)
B1.23.2	IgG2b/*eBiosciences*	β_2_M bound, fully assembled HLA-B and -C molecules. Precise epitope unknown	HLA-B and HLA-C	(52)
6A4	IgG1/*Hybridoma*	β_2_M bound, fully assembled HLA-B and -C molecules. Precise epitope unknown	HLA-B and HLA-C	(53)
HC10	IgG2a/*Hybridoma*	Free heavy chain of HLA class I. Residues 57–62, specifically residue 60 in the α1 domain in HLA-C. The epitope is blocked by peptide binding	HLA-B and HLA-C and some HLA-A (A10, A28, A29, A30, A31, A32, A33)	(54, 55)
DT9	IgG2b/*Hybridoma*	Fully assembled HLA-C and HLA-E bound to β_2_M. Precise epitope unknown	HLA-C and HLA-E	(56)
L31	IgG1/*MediaParma*	Free heavy chain of HLA-C. Residues 66–68, with F or Y at position 67	Most HLA-C and a few HLA-B (B08, B07, B35, B51, B54, B56)	(57)

#### Functional Assay with Primary NK Cells Including Subsequent Multicolor Immunofluorescence Staining

Natural killer cells were stimulated with medium alone or with IL-12 (10 ng/ml, R&D systems) and IL-15 (50 ng/ml, R&D systems) for 12 h. The following day, target cells were added at an E:T ratio of 1:1 or 10:1 (58). Cytokine-stimulated NK cells in medium alone, or cocultured with K562 cells (a gold standard NK cell target cell line, devoid of MHC class I) served as a positive control for function when HCMV-infected HFFFs were investigated for their capacity to trigger primary NK cells. After 1 h of coculture, GolgiPlug (Brefaldin A, BD Bioscience) and GolgiStop (Monensin, BD Bioscience) were added to the wells according to manufacturer’s instructions, and incubated for an additional 4 h. Non-adherent cells were subsequently transferred to V-bottom well plates where multicolor immunofluorescence staining was performed (32). The following conjugated monoclonal antibodies were used: anti-KIR2DL3 FITC (REA147, Miltenyi), anti-NKG2A APC (Z199, Beckman Coulter, Brea, CA, USA), anti-CD107a APC-H7 (H4A3, BD Bioscience), anti-KIR3DL1 Brilliant Violet 421 (DX9, BioLegend), biotinylated anti-KIR2DL1 (143211, R&D Systems), anti-CD56 ECD (N901, Beckman Coulter), anti-CD3 PE-Cy5 (UCHT-1, BioLegend), and anti-KIR2DL1/S1 PE-Cy7 (EB6, Beckman Coulter). Briefly, the cells were stained with the primary antibody cocktail for 20 min. The anti-KIR2DL1/S1 antibody was added directly to the wells (dilution 1:20) for 10 min incubation. Following washing, the secondary antibody cocktail containing Live/Dead Aqua (Invitrogen, Carlsbad, CA, USA) and Qdot-605-conjugated Streptavidin (Invitrogen) was incubated for 20 min. Cells were next washed and fixed.

Data acquisition for the cellular and immunofluorescence cell-surface staining experiments was performed on FACScan (Department of Pathology), FACSCalibur or Accuri C6 flow cytometer (NIHR Cambridge BRC Cell Phenotyping Hub, BD Bioscience) depending on the experiment. The data acquisition of the multicolor primary NK cell staining experiments was performed on a BD LSR Fortessa flow cytometer, equipped with 4 lasers and 13 PMTs (Cambridge Stem Cell Institute). Flow cytometry data were analyzed using FlowJo software (TreeStar, OR, USA).

### Viruses

Human cytomegalovirus strains TB40/E [isolated from a throat wash of a bone marrow transplant recipient (59)], AD169 (ATCC VR-538) and Merlin (a gift from Richard Stanton, University of Cardiff, UK) were grown, concentrated, and titrated as described previously (60). Confluent plates of HFFFs or DFs were infected with concentrated virus (TB40/E, AD169, Merlin) at a multiplicity of infection (MOI) as indicated in the experiment or with diluted (1:10) supernatant-containing virus (TB40/E-derived clones). The virus preparations were diluted in complete DMEM to obtain the required MOI. After 1 h of infection at room temperature, the cells were washed with PBS, and fresh complete DMEM was added. A coculture experiment was performed, or the cells were harvested for cell-surface staining, or western blotting, 24, 48, and/or 72 h p.i. as indicated in the experiment. Time points were chosen to reflect the temporal cascade of expression of HCMV viral proteins (immediate early, early, and late genes). Late genes, which are structural viral components, are last expressed at ~72 h post infection (p.i.) (61). UV inactivation of the virus was obtained by exposing the virus 30 min to UV light of a 30 W germicidal lamp.

### Plaque Purification Assay

Human fetal foreskin fibroblasts were infected with serially diluted HCMV strain TB40/E. After 1 h of infection at room temperature, the virus-containing medium was replaced with 5 ml per well of 2% agarose mixed 1:1 with two times concentrated DMEM medium (Millipore, Bedford, MA, USA) containing 20% FCS, 200 U/ml penicillin, 200 μg/ml streptomycin, 8 g/L sodium bicarbonate (GE Healthcare), and 1 mM of sodium pyruvate (Sigma-Aldrich). After 3 weeks, plaques (areas of dead cells) were visible by eye. Well-separated plaques, representing different viral clones of the TB40/E wild-type strain, were picked using a glass Pasteur pipette by removing the agar and plaque as a plug. The agarose plug containing viral clones were disrupted and added to freshly plated HFFFs in 96-well flat-bottom culture plates to expand the viral clones. Selected clones were expanded up to a 75 cm^2^ culture flask. After 100% infection was reached, as determined visually by microscopy, the supernatant-containing virus was harvested and stored at −80°C. Fresh complete DMEM was added to the flasks and further harvests were carried out every 72 h until approximately 90% cell lysis was visually determined. All the harvested supernatant was pooled, spun at 2,000 rpm to remove cell debris, aliquoted, and stored at −80°C.

### Western Blot

Cells were washed with PBS and lysed in lysis buffer [1% Triton X-100, 20 mM Tris–HCl (pH 7.4), 150 mM NaCl, 1 mM EDTA, 5 mM MgCl_2_, protease inhibitors (Roche, Mannheim, Germany), and 1 mM Phenylmethanesulfonyl fluoride (PMSF, Sigma-Aldrich) and 1.25 mg/ml N-ethylmaleimidine (Sigma-Aldrich)] for 20 min at 4°C. Debris and nuclei were removed by centrifugation at 13,000 rpm (centrifuge HAWK 15/05, MSE), and a bicinchoninic acid (BCA) assay (Thermo Scientific) was used to determine protein concentrations.

Supernatants of TB40/E-derived clones were pelleted by centrifuging at 15,000 rpm for 2 h at 4°C using an Avanti J-25 Ultracentrifuge (Beckman Coulter). The virus pellets were gently washed with PBS, and 200 μl of lysis buffer was added to lyse the particles. Lysates from positive and negative virus particles were paired based on approximately the same infectivity titer. Virus titers were calculated by TCID_50_.

Total cell or virus particle lysates were loaded onto a 10–13% SDS-PAGE gel, and proteins were transferred onto Immobilon-P PVDF membranes (Millipore). The membranes were blocked 1 h in PBS, 5% dried milk, and 0.05% Tween 20 at room temperature. The membranes were incubated overnight at 4°C or 3 h at room temperature with primary antibodies [anti-pp28 (Abcam), L31, HC10, anti-I.E.1 (Merck Millipore, Billerica, MA, USA), anti-Calnexin (Enzo Life Sciences, Farmingdale, NY, USA), and anti-Flag (M2, Sigma-Aldrich)] at concentration of 0.1–0.2 μg. The membranes were washed thoroughly, and polyclonal HRP-conjugated goat anti-mouse IgG or goat anti-rabbit IgG (Dako, 1:4,000 dilution) secondary antibody was added for 30 min at room temperature. Chemiluminescence was performed according to the manufacturer’s instructions using ECL Prime (GE Healthcare) or home-made ECL (62).

### Establishment CRISPR-CAS9 Knockout (KO) for β_2_M in HFFFs

*Streptococcus pyogenes* CAS9 and short-guide RNA (sgRNA) were expressed in separate lentivirus constructs: pHRSIN containing the SFFV promoter, FLAG tag, nuclear localization signals (NLS), CAS9 and pGK Hygro (kind gift from Lehner’s group, CIMR, University of Cambridge), and pKLV-containing U6 promoter, sgRNA (modified *Bbs*I), pGK Puro, 2A, and BFP tag [Addgene plasmid #50946; created by Kosuke Yusa, Wellcome Trust Sanger Institute, Cambridge, UK (63)]. The pKLV construct containing sgRNA-targeting β_2_M was a gift from Dick van den Boomen (CIMR, University of Cambridge), using the sgRNA sequence 5′-GGCCGAGATGTCTCGCTCCG-3′.

The pHRSIN and pKLV lentivirus constructs (6 μg) were packaged together with pMDG and pCMV9.81 packaging vectors (4 μg) in HEK 293T cells in 75 cm^2^ culture flasks using Opti-MEM™ and GlutaMax™ media (Gibco), and *Trans*IT^®^ Transfection reagent (Mirus, Madison, WI, USA). After 48 h, supernatant-containing lentiviral particles were used to transduce HFFFs in 75 cm^2^ culture flasks by adding Polybrene (8 ng/ml). The transduced cells were selected with 200 μg/ml hygromycin and/or 2 μg/ml puromycin and grown until a confluent 75 cm^2^ culture flask was reached. Cells negative for total HLA class I were purified by surface staining using W6/32 antibody, respectively, followed by single-cell sorting using the FACS sorter. The CAS9 is FLAG-tagged and a HRP-conjugated anti-M2 FLAG antibody was used to detect it by western blot.

### Statistical Analysis

Non-parametric one-way analysis of variance (ANOVA) using the Kruskal–Wallis test and Dunn’s multiple comparisons test was used to determine the statistical significance. In these tests, a *p* value of less than 0.05 was considered significant (*p < 0.05, **p < 0.01). The tests were done with GraphPad Prism version 6.00 (GraphPad software).

## Results

### The Function and Specific Recognition of KIR2DS1 by Reporter Cells

**Figure 1:**
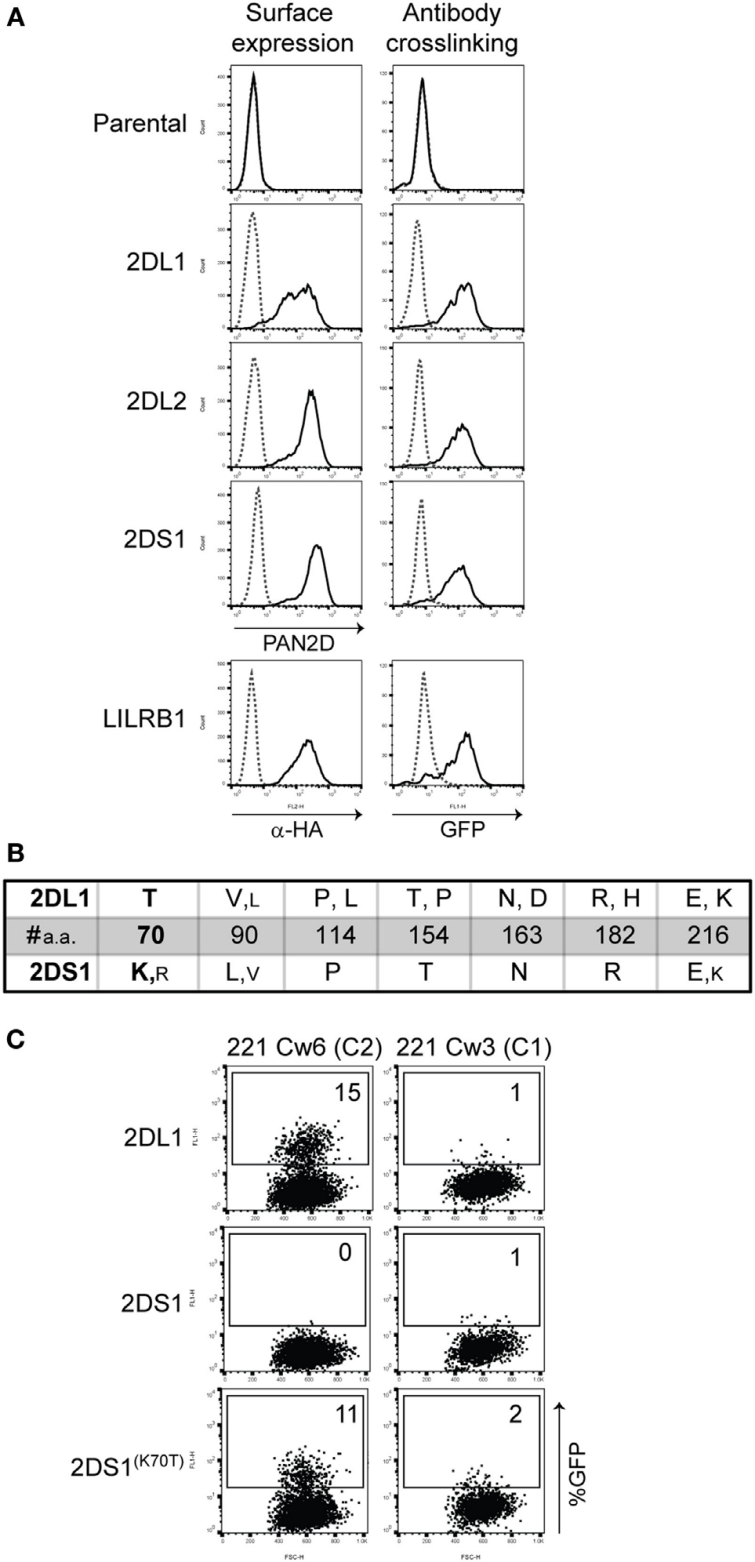
**2B4 reporter system for activating killer cell Ig-like receptor (KIR)**. **(A)** Cell-surface expression on the indicated reporter cells was measured by flow cytometry after staining the cells with PAN2D (clone NKVFS1, recognizes all KIRs) or anti-HA antibody (black line, left panel). Parental 2B4 cells containing only the adaptor protein were used as a negative control. To test the function of these reporter cells, plate-bound PAN2D (clone NKVFS1) or anti-HA (LILRB1 reporter only) antibody crosslinking (black line) was used to engage KIR or LILRB1 molecules on the reporter cells during an overnight incubation (right panel). IgG1 antibody was used as isotype control (dotted line). **(B)** Table depicting amino acid positions of polymorphic sites in the extracellular domain of KIR2DL1 and -2DS1 alleles. Large letters indicate amino acids present in the majority of alleles, and small letters indicate amino acids that are present in few alleles. At position 70, a unique amino acid in KIR2DL1 and -2DS1, threonine (T) and lysine (K), respectively, was found. Allele sequences were aligned using the alignment tool in the *IPD-KIR database*. **(C)** KIR2DS1^(K70T)^ was transfected into the 2B4 reporter system and an overnight coculture was performed using KIR2DL1, -2DS1, or -2DS1^(K70T)^ reporter cells together with 721.221 cells expressing human leukocyte antigen (HLA)-Cw3 (C1) or HLA-Cw6 (C2). The next day green fluorescent protein (GFP) expression was measured by flow cytometry. The E:T ratio of the coculture was 1:3.

Since KIR2DS1 binds C2-HLA-C only weakly, we aimed to investigate what might influence stronger binding. To accomplish this, we used a specifically designed mouse 2B4 T cell hybridoma carrying an NFAT-GFP reporter system, similar to that described by Arase et al. (43). KIR2DS1 was transduced into these 2B4 reporter cells, together with a modified adaptor protein. Once KIR2DS1 binds its cognate ligand, a signaling cascade is triggered through the adaptor protein, which then transcribes NFAT, resulting in GFP expression. KIR2DL1, -2DL2, and LILRB1 reporter cells were also generated. We confirmed that the reporter cells were constructed successfully by staining the surface expression of the different receptors with the relevant antibodies and by engaging the reporter cells with relevant plate-bound antibodies (Figure 1A).

There are seven amino acid differences between KIR2DL1 and -2DS1 alleles, including the threonine to lysine in KIR2DL1 and -2DS1 at position 70, respectively (Figure 1B). We wanted to investigate whether substituting the lysine to a threonine at position 70 in KIR2DS1 would result in the activation of the reporter cells as a result of the interaction with C2-HLA-C, as demonstrated previously by Biassoni and colleagues using Fc proteins (25). We made the KIR2DS1^(K70T)^ reporter cell and cocultured these cells with 721.221 transfected with HLA-C03:02 (C1) or -C06:02 (C2) (221-Cw3 or 221-Cw6). Indeed, both KIR2DL1 and KIR2DS1^(K70T)^ reporter cells were activated after coculture with 221-Cw6 (15 and 11% GFP-positive cells, respectively) and not with 221-Cw3 (Figure 1C). This confirms the observations made by Biassoni et al. in our cellular reporter system and shows that the reporter cells are functional, specific, and sensitive.

### KIR2DS1 Reporter Cells Do Not Recognize Conventional HLA Class I

**Figure 2:**
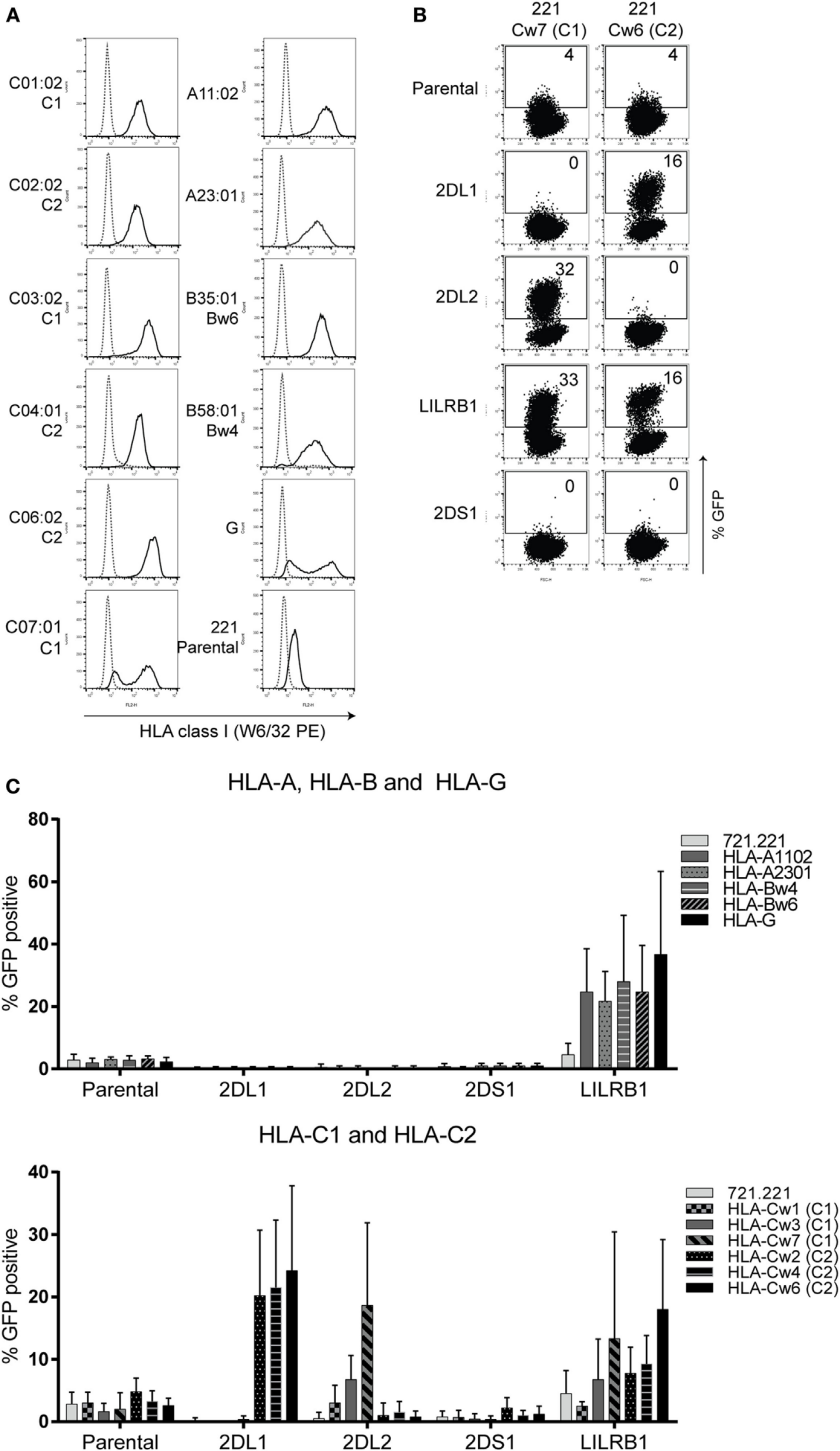
**KIR2DS1 reporter cells are not activated by conventional human leukocyte antigen (HLA) class I molecules**. **(A)** The 721.221-HLA transfectants were stained with W6/32 (black line), and measured by flow cytometry. IgG2a antibody was used as isotype control (dotted line). **(B)** Dot plots of a selection of representative data from the same experiment. A coculture of the indicated reporter cells together with 721.221 cells containing HLA-C07:01 (221 Cw7, C1) or HLA-C06:02 (221-Cw6, C2) is shown. GFP expression was determined by flow cytometry. The E:T ratio was 1:3. **(C)** The data are depicted as the mean ± SD of individual samples collected from five independent experiments.

To verify whether KIR2DS1 reporter cells are activated by conventional HLA class I molecules, cocultures were performed using the KIR2DS1 reporter cells along with appropriate controls. The cocultures were done using a range of 221 cells transduced with different HLA molecules: HLA-A11:02, -A23:01, -Bw6 (B35:01), -Bw4 (B58:01), -G, -C01:02 (C1), -C02:02 (C2), -C03:02 (C1), -C04:01 (C2), -C06:02 (C2), and -C07:01 (C1). HLA expression levels on these 221 cells were confirmed by staining with W6/32 antibody (Figure 2A). KIR2DS1 reporter cells were not activated in cocultures with different 221-HLA-C or other 221-HLA class I cells. However, the positive controls KIR2DL1, -L2, and LILRB1 were activated after engaging their documented ligands (Figures 2B,C). As expected, KIR2DL1, -L2, and LILRB1 reporter cells were differentially activated, depending on the HLA-C allele they engaged. For instance, the KIR2DL2 reporter cell was weakly activated after binding HLA-C01:02 (<5% GFP-positive cells) compared to HLA-C07:01 (>20% GFP cells), as depicted in Figure 2C, lower panel. This is in line with binding studies using Fc proteins and HLA-coated beads (11, 64).

**Figure 3:**
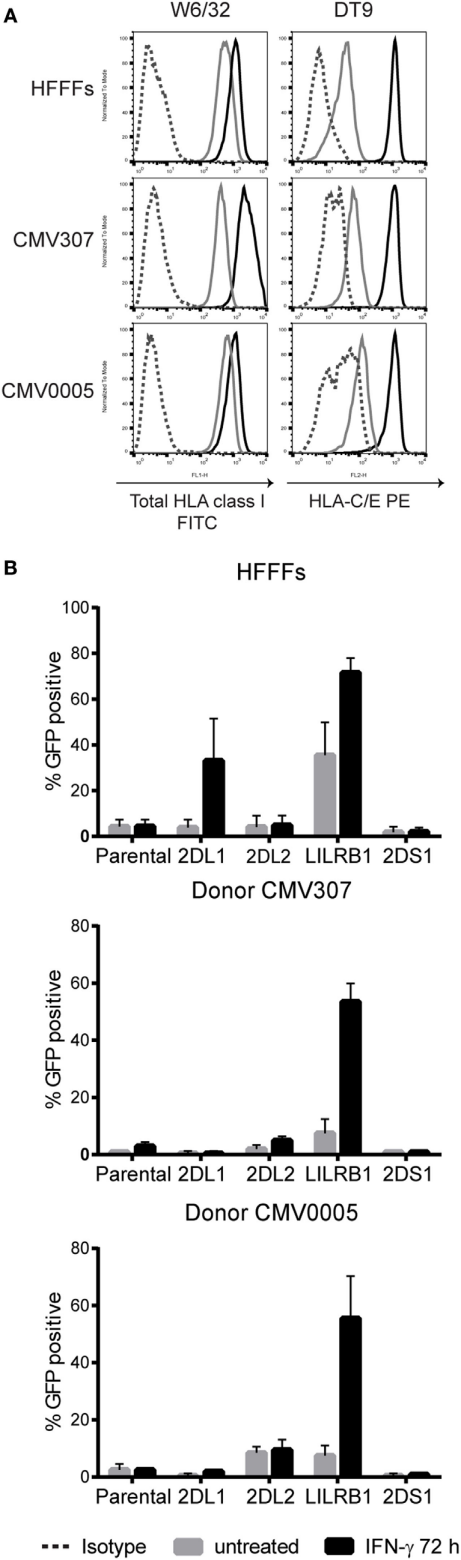
**KIR2DS1 reporter cells are not activated by interferon (IFN)-γ stimulated human fetal foreskin and primary dermal fibroblasts of healthy individuals**. **(A)** Total human leukocyte antigen (HLA) class I (W6/32) and HLA-C/-E (DT9) expression levels of untreated and IFN-γ stimulated HFFFs (C2/C2) and DFs of donor CMV307 (C1/C1) and CMV0005 (C1/C2) were measured by flow cytometry. Gray = untreated, black = IFN-γ stimulated (500 U/ml, 72 h) and dotted line = isotype control. **(B)** The untreated and IFN-γ-stimulated fibroblasts were cocultured overnight with the indicated reporter cells, and green fluorescent protein (GFP) expression was measured by flow cytometry. Three independent experiments are depicted in the bar graphs as the mean ± SD of individual samples.

In our hands, the KIR2DS1 reporter cells did not recognize endogenously expressed HLA class I molecules on 221 cells. To investigate this further, they were cocultured with primary cells: HFFFs, CMV307, and CMV0005 DFs, which were stimulated with or without IFN-γ for 72 h. Before the coculture experiments, HLA class I surface expression levels of untreated and IFN-γ stimulated cells were measured. The primary fibroblasts all expressed high levels of folded HLA class I and HLA-C/-E molecules, revealed by W6/32 and DT9 antibody staining, respectively. These HLA class I levels were further increased after 72 h of IFN-γ stimulation (Figure 3A). KIR2DS1 reporter cells remained GFP-negative when cocultured with the different untreated or IFN-γ stimulated primary fibroblasts (Figure 3B). By contrast, LILRB1 reporter cells were activated in all coculture experiments with untreated cells and were further activated in settings with IFN-γ stimulated cells. KIR2DL2 and KIR2DL1 reporter cells were activated, in some conditions only minimally, depending on the particular HLA-C alleles expressed by the cells (Figure 3B). KIR2DS1 reporter cells were similarly unresponsive to tumor cell lines including HeLa, Meljuso, Caski, and JEG-3 cells (data not shown). In conclusion, KIR2DS1 reporter cells were not activated after engaging with conventional C2-HLA-C molecules.

### KIR2DS1 Reporter Cells Bind a Ligand on HFFFs Infected with Specific HCMV Clones

Since KIR2DS1 reporter cells were not activated by conventional HLA molecules, we considered that KIR2DS1 might recognize a pathogen-induced ligand. Several studies have suggested a role for aKIR in HMCV infection (44–47). We therefore investigated whether the KIR2DS1 ligand might be upregulated after HCMV infection. HFFFs were infected with the HCMV TB40/E strain for 24, 48, and 72 h and cocultured with the panel of reporter cells. HFFFs were used because they support full lytic HCMV infection (65) and express HLA-C02:02 and -C04:01, both C2-HLA-C.

#### HFFFs Infected with the TB40/E Wild-Type Strain Express a Ligand for KIR2DS1

**Figure 4:**
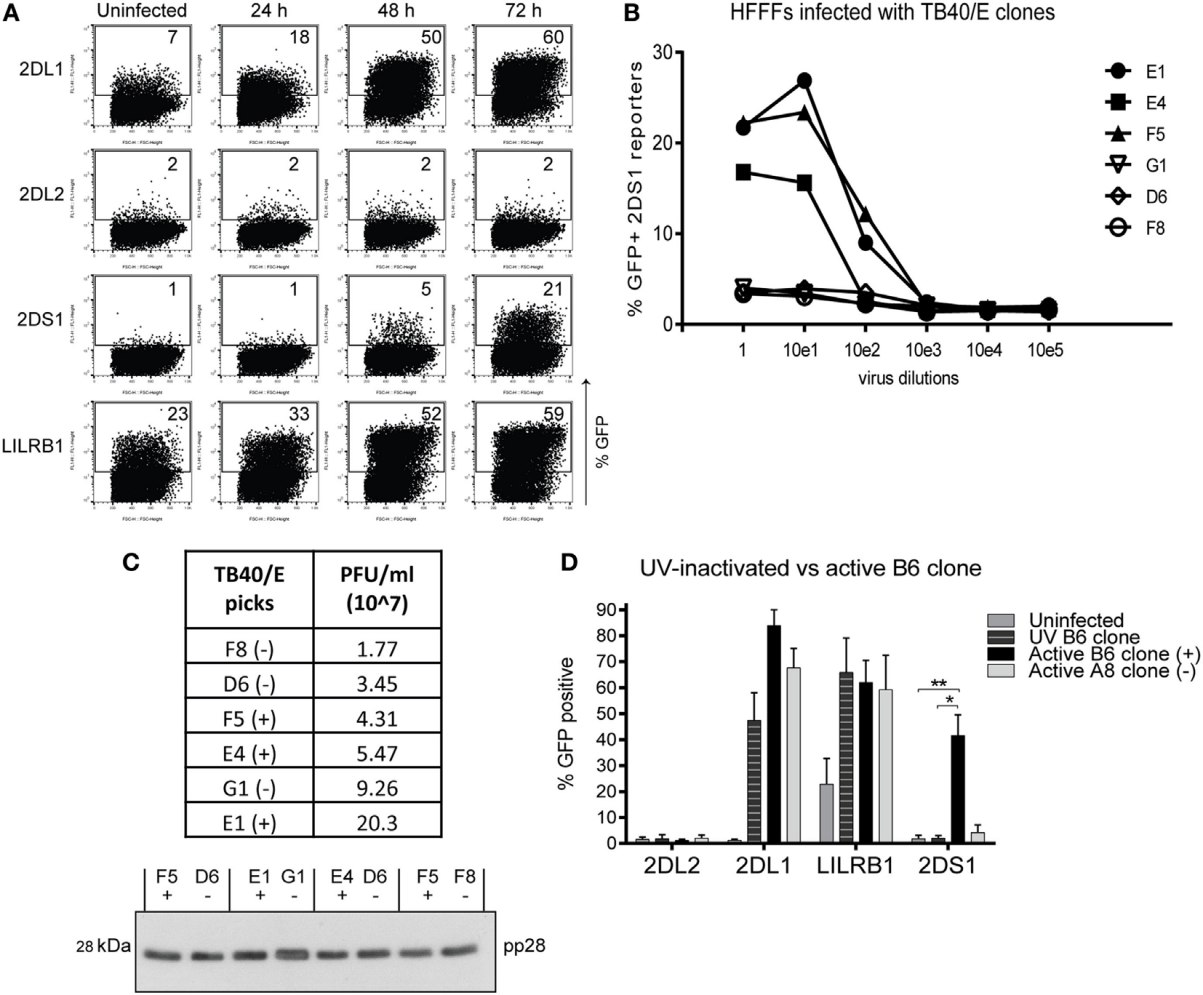
**The KIR2DS1 reporter cell recognizes a ligand on human fetal foreskin fibroblasts (HFFFs) infected with specific human cytomegalovirus (HCMV) strains**. **(A)** Uninfected HFFFs and 24, 48, and 72 h infected HFFFs with HCMV TB40/E strain with an MOI of 10 were cocultured overnight with the reporter cells as indicated. A representative experiment of two independent time course cocultures is shown. **(B)** HFFFs were infected with six different TB40/E-isolated clones (E1, E4, F5, G1, D6, and F8) in 10-fold serial dilutions for 72 h and cocultured with KIR2DS1 reporter cells. A representative experiment from over three independent experiments is depicted. **(C)** The infectivities of three positive and three negative TB40/E clones were calculated by TCID_50_ assay and are depicted in the table (virus titers in PFU/ml). Viral particles were isolated from the supernatant, lysed, and loaded onto a 12% SDS-PAGE gel for western blotting with anti-pp28 antibody. Positive and negative clones were paired based on approximately the same infectivity. Equal amounts of lysate from these pairs were loaded onto the gel. (−) indicates a negative clone, (+) positive clone. **(D)** HFFFs were stimulated with UV-inactivated TB40/E, infected with the positive B6 or negative A8 clone for 72 h. Forty-eight hours p.i. KIR2DL2 (negative control), KIR2DL1, LILRB1 (positive control), and KIR2DS1 reporter cells were added. After an overnight coculture, the GFP expression was measured using flow cytometry. The data are depicted as the mean ± SD of individual samples collected from five independent experiments. *p < 0.05 and **p < 0.01 are calculated by non-parametric one-way ANOVA using the Kruskal–Wallis test and Dunn’s multiple comparisons test.

In coculture, KIR2DS1 reporter cells increased in GFP positivity from 1% (uninfected and 24 h) to 5% (48 h) and to 21% positive cells (72 h) (Figure 4A). This indicates that KIR2DS1 recognized a ligand, which was detected by the reporter cells 48 h p.i. with HCMV. In addition, there was increased triggering of both KIR2DL1 and LILRB1 reporter cells over time (Figure 4A). This was of interest as HCMV downregulates HLA class I molecules (66–71), and a decrease in KIR2DL1 reporter activation over infection time was expected. The increase in the LILRB1 reporter cell activation might be explained by the recognition of UL18 protein, which HCMV produces as an immune evasion strategy (72, 73). Subsequently, in a total of 10 KIR2DS1 reporter cell coculture experiments with HFFFs infected with HCMV TB40/E strain were done, demonstrating the reproducibility of the KIR2DS1 reporter cell activation after infection (Figure S1 in Supplementary Material). The KIR2DS1 ligand was only expressed on HFFFs infected with the TB40/E clinical strain and not with other strains tested, such as Merlin (another clinical strain) and AD169 (laboratory strain with deletion in the ULb’ region) (data not shown).

#### Specific TB40/E Clones Activate the KIR2DS1 Reporter Cells

Tomasec and colleagues isolated two viral clones, called Lisa and Bart, from the TB40/E strain, which had different functional properties (74). We wanted to investigate whether different viral clones were also present in our TB40/E strain. Multiple viral clones were isolated using a plaque purification assay. Twelve clones were selected and used to infect HFFFs followed by coculture with KIR2DS1 reporter cells. Six clones (B6, D7, E1, E4, E5, and F5) activated KIR2DS1 reporter cells (referred to as positive clones in what follows), while the other “negative” clones (A6, A8, D6, F2, F8, and G1) did not. Figure 4B depicts a representative coculture experiment where HFFFs were infected with six randomly selected clones in a 10-fold serial dilution for 72 h and then cocultured with KIR2DS1 reporter cells. The reporter cells were highly activated by HFFFs infected with the positive clones; E1, E4, and F5. They were not activated after infecting with negative clones; G1, D6, and F8 (Figure 4B). Infecting HFFFs with the positive clones resulted in a higher percentage of GFP-positive KIR2DS1 reporter cells (ranging from 16% by E4 clone to 50% by B6 clone) compared to the wild-type TB40/E strain (ranging from 4 to 21%, Figure S1 in Supplementary Material), indicating that the positive clones induced the KIR2DS1 ligand more efficiently. This is in line with the idea that the parental TB40/E strain contains a mixture of positive and negative viruses with respect to KIR2DS1 ligand expression.

Human cytomegalovirus infection indeed led to reduction in total HLA class I expression on most HFFFs as monitored by binding of W6/32 antibody and this was true of both positive and negative clones (Figure 6A). This indirectly indicates that the positive and negative clones were equally infectious and consistently infected over 90% of the HFFFs. In addition, the differential response of the KIR2DS1 reporter cells to the positive and negative clones was not due to differences in overall viral particle numbers of the different clones. This was demonstrated by comparing the infectivity (functional virus particles) with the total number of viral particles (functional and empty/non-functional particles) by detecting a structural tegument protein pp28 by western blot after pairing positive and negative virus clones based on approximately the same infectivity titer (Figure 4C).

#### KIR2DS1 Ligand Is Only Expressed on HFFFs after Infecting with Infectious Virus

Within the investigated infection timeframe, non-infected cells could be refractory for HCMV infection, yet exposed to pro-inflammatory cytokines, such as type I interferons (IFNs), which subsequently induce HLA class I surface expression. It was therefore critically important to investigate whether KIR2DS1 reporter cells were activated by the infected cells or by the surrounding non-infected cells. To examine this, TB40/E viruses were exposed to UV light for 30 min to inactivate the virus. After exposure, viral particles will be present and able to enter the cell, but the viral genes will be inactivated and will not be transcribed. Virus inactivation was confirmed by immunohistochemistry staining of Immediate Early 1 (I.E.1) viral proteins on the treated HFFFs, as shown in Figure S2 in Supplementary Material. These UV-inactivated viruses are called “UV virus.”

After stimulating HFFFs with UV B6 clone and infecting with the positive B6 and negative A8 clones for 72 h, a coculture with parental, KIR2DL1, LILRB1, and KIR2DS1 reporter cells was performed. KIR2DS1 reporter cells were significantly activated in coculture with the active B6 clone-infected HFFFs, but not with UV B6-stimulated HFFFs nor in any other conditions, as shown in Figure 4D. By contrast, the KIR2DL1 reporter cells were highly activated after encountering UV B6-stimulated HFFFs, as well as with B6 clone-infected HFFFs (Figure 4D). Total HLA class I and HLA-C/-E cell-surface levels using W6/32 and DT9 antibodies were assessed and both HLA-A, -B, -C and HLA-C/-E surface expression levels were highly increased on UV virus-stimulated HFFFs (Figure 6). Since fibroblasts are known to produce IFN-α and -β (75, 76), it was expected to see such an increase in HLA class I cell-surface levels, after exposure to viral particles. This is the host response to HCMV particles without the interference of HCMV genes downregulating HLA class I molecules.

In conclusion, from the different HCMV strains tested, only the TB40/E strain activated KIR2DS1 reporter cells after infecting HFFFs. Furthermore, the TB40/E strain consists of different virus clones and these virus clones differentially activated KIR2DS1 reporter cells. Since the clones have comparable amounts of functional viral particles, the levels of activation were not governed by the number of viral particles. We may also conclude that active viral gene expression is necessary to induce the KIR2DS1 ligand.

### Primary, Single-Positive KIR2DS1 NK Cells Are Only Activated in Coculture when HFFFs Are Infected with Specific HCMV Clones

After establishing that KIR2DS1 reporter cells recognize a ligand on HFFFs infected with specific clones, we asked whether primary NK cells would also interact with these infected cells. Coculture experiments were performed using freshly isolated peripheral blood NK cells from healthy individuals. NK cells expressing KIR2DS1 are hyporesponsive, if the donor is homozygous for C2-HLA-C (32). We therefore chose donors that were C1-HLA-C homozygous, bearing fully functional KIR2DS1-positive NK cells. General NK cell functionality was verified using IL-12/15 primed NK cells cocultured with the prototypic HLA class I negative NK cell target cell line K562 (positive control), or with no target cells (negative control). The KIR^−^ NK cell subset represents the KIR-independent activation of NK cells, which is the background NK cell activation in this experiment.

**Figure 5:**
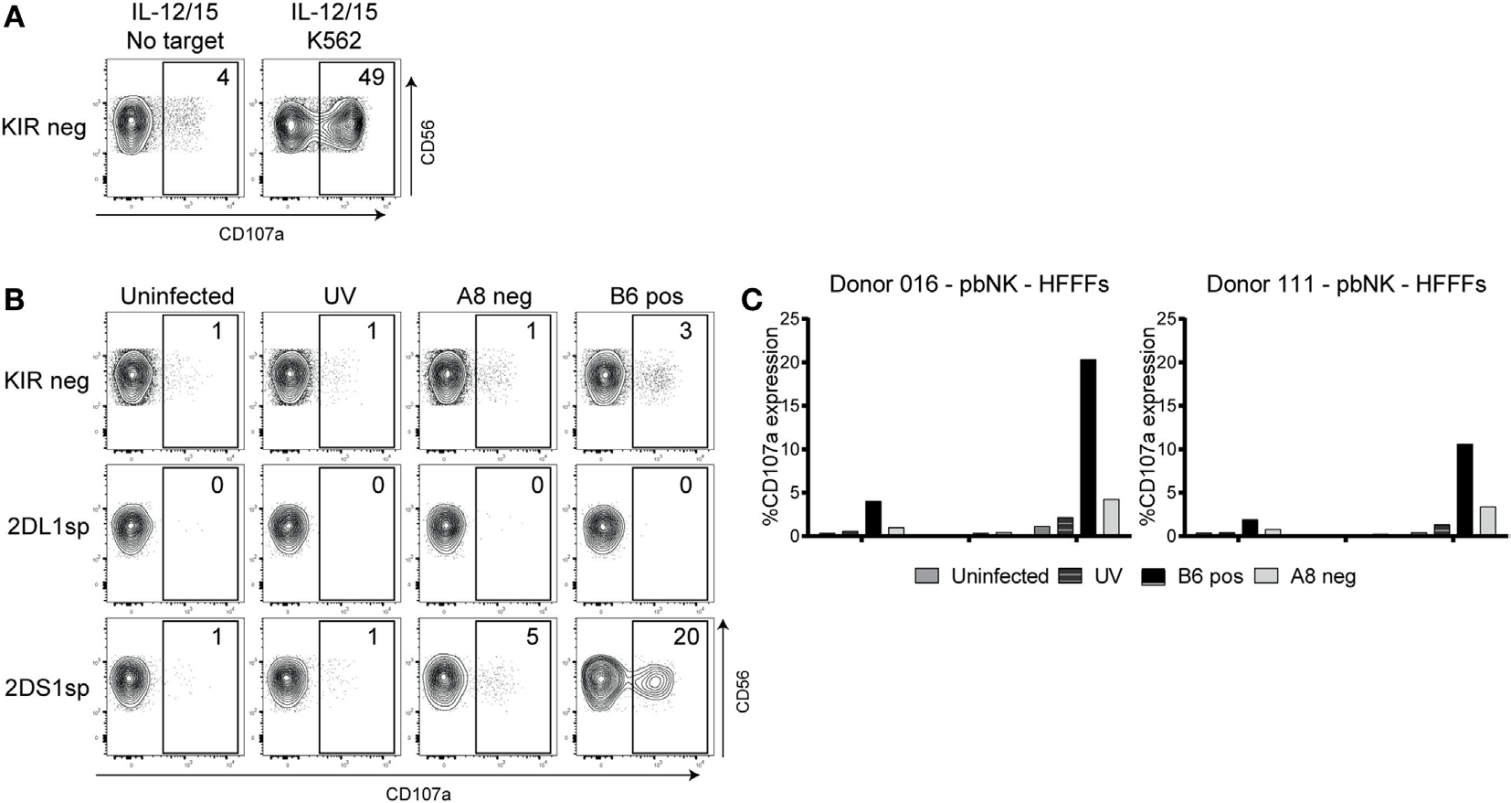
**Primary natural killer (NK) cells degranulated after coculture with human fetal foreskin fibroblasts (HFFFs) infected with the positive B6 TB40/E clone**. **(A)** After NK cell isolation, the NK cells of donor 016 were rested or stimulated with IL-12 and IL-15 overnight. IL12/IL15-stimulated NK cells were cocultured with and without K562 cells. **(B)** Unstimulated NK cells were cocultured with uninfected HFFFs, UV B6 clone-stimulated HFFFs (UV), negative A8 clone- (A8 neg), and positive B6 clone-infected HFFFs (B6 pos). After harvesting, the NK cells were stained with antibodies targeting different surface markers and the degranulation marker CD107a. NK cells from the KIR-negative (KIR), KIR2DL1 single-positive (2DL1sp), and KIR2DS1 single-positive (2DS1sp) subsets are shown. The percentage of CD107a expression is depicted. The NK subsets are grouped on NKG2A^−^ NK cells (A^−^). **(C)** The same coculture experiment with NK cells of donor 016 is represented in a bar graph (left) including coculture data of NK cells of donor 111 (right). The percentage of CD107a expression is shown. All cocultures were performed for 5 h at an E:T ratio of 1:1. A representative data from three independent coculture experiments are shown. The other two independent experiments are illustrated in Figure S4 in Supplementary Material.

Forty-nine percent of the cytokine-primed KIR^−^ NK cells degranulated after NK cells of donor 016 encountered K562 cells, and degranulation of 4% was observed in culture without target cells, indicating that the NK cells are functional, and little background activation was observed (Figure 5A). In the same experiment, rested NK cells (without cytokine stimulation) were cocultured with HFFFs. NKG2A^−^ cells were separated into KIR-negative (KIR^−^), KIR2DL1 single-positive (2DL1sp), and KIR2DS1 single-positive (2DS1sp) NK cells. The gating strategy is described in Figure S3 in Supplementary Material. No response (1% CD107a expression) was seen from 2DS1sp NK cells cocultured with uninfected or UV B6 clone-stimulated HFFFs (Figure 5B). Similarly, minimal functional response from 2DS1sp NK cells was observed after coculture with HFFFs infected with the negative A8 clone (5% CD107a expression). Notably, 2DS1sp NK cells engaging HFFFs infected with the positive B6 clone showed 20% CD107a expression (Figure 5B). Only a slight background activation of 3% was observed in the KIR^−^ population in coculture with positive B6 clone-infected HFFFs. In all the other conditions, no degranulation in the KIR^−^ population was observed. Furthermore, in every condition, including the positive B6 clone condition, no degranulation of 2DL1sp NK cells was observed (Figure 5B). The experiment was repeated with isolated NK cells of donor 111, and these NK cells responded similarly, though slightly weaker, to the different types of treated HFFFs (Figure 5C). These results were reproducible in two independent coculture experiments using either isolated NK cells (Figure S4A in Supplementary Material) or PBMCs (Figure S4B in Supplementary Material).

In conclusion, similar to the reporter cells, primary NK cells expressing KIR2DS1 recognize a ligand on HFFFs infected with specific clones of the TB40/E strain.

### Specific HCMV Clones Are Less Effective in Targeting HLA-C

After confirming that the KIR2DS1 ligand is expressed on HFFFs infected with positive clones of HCMV, different HLA class I surface levels of uninfected HFFFs, UV B6 clone-stimulated HFFFs, and both positive and negative clone-infected HFFFs were compared. Surface expression of HLA-E (3D12), HLA-C/-E (DT9), total HLA class I (W6/32), HLA-A11, HLA-Bw6, total free heavy chain (FHC) of HLA class I (HC10), and FHC of HLA-C (L31) was analyzed. The antibodies used are listed in Table 1 with a description of their specific recognition patterns and are discussed below.

#### Free Heavy Chain of HLA-C and Assembled HLA-C Remain on the Cell Surface of HFFFs Infected with Positive Clones

**Figure 6:**
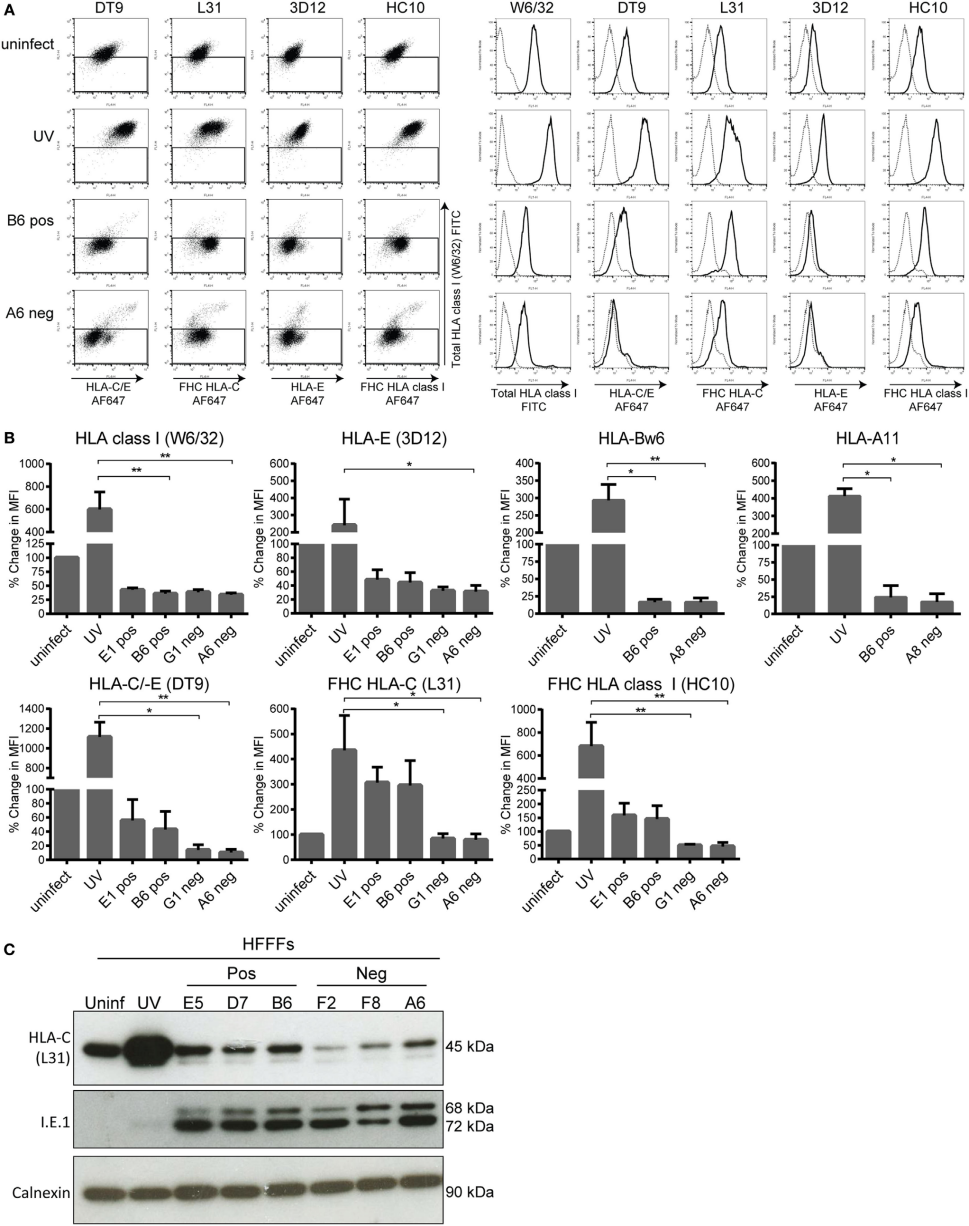
**Human leukocyte antigen (HLA) class I surface and total HLA-C protein expression of infected human fetal foreskin fibroblasts (HFFFs)**. **(A)** Dot plots (left) and histograms (right) from the same representative experiment are shown. The HFFFs infected with the indicated positive or negative clones and the UV B6 clone-stimulated HFFFs (UV) were treated for 72 h. The indicated treated HFFFs were stained subsequently with W6/32 (total HLA class I), DT9 (HLA-C/-E), 3D12 (HLA-E), L31 (FHC HLA-C), and HC10 (FHC HLA class I) antibodies (black line). Cells stained with the appropriate isotype control (dotted line) were included. **(B)** Collection of up to four independent cell-surface staining experiments is depicted in bar graphs as the mean ± SD of individual samples. Anti-Bw6 and anti-A11 antibodies were also included. The percentage change in MFI is depicted with the uninfected condition at 100%. *p < 0.05 and **p < 0.01 are calculated by non-parametric one-way ANOVA using the Kruskal–Wallis test and Dunn’s multiple comparisons test. **(C)** Total lysate of uninfected, UV B6 clone-stimulated (UV), positive clones- (E5, D7, B6), and negative clones (F2, F8, A6)-infected HFFFs were loaded onto a reduced 10% SDS-PAGE gel. The membrane was blotted with the L31, anti-I.E.1 viral protein (measure of infection) and anti-calnexin (loading control) antibodies.

Positive and negative clones both reduced total HLA class I, HLA-E, HLA-Bw6, and HLA-A11 cell-surface levels on infected HFFFs compared to the UV B6 clone-stimulated HFFFs (*p* < 0.05 and *p* < 0.01, Figures 6A,B). However, using antibodies to FHC forms of HLA, such as HC10 and, in particular, L31 staining was higher in HFFFs infected with the positive clones compared to the negative clones. FHC HLA-C levels, as detected by L31, were close to the levels of UV B6-stimulated HFFFs, indicating that the positive clones did not effectively downregulate the FHC HLA-C surface levels (Figures 6A,B). This difference observed in L31 staining was reproducible in every experiment performed (*n* = 11) and in an experiment where FHC HLA-C surface levels were compared in HFFFs infected with 12 additionally isolated clones comprising six positive and six negative clones (Figure S5A in Supplementary Material). Similar differences between the clones were observed with HC10 staining (FHC of HLA class I molecules, Figures 6A,B). This difference could be due to the antibody detecting the elevated FHC of HLA-C specifically. However, this remains uncertain, because specific antibodies against FHC of HLA-A and -B are not available.

The DT9 staining (detects conformational HLA-C/-E containing β_2_M) was slightly weaker and less consistent than L31, but similar differences between the clones were measurable (Figures 6A,B). Since DT9 antibody cross-reacts with HLA-E (56), an anti-HLA-E monoclonal antibody (3D12) was included. HLA-E surface expression in general was very low on HFFFs (Figure 6A) and no difference was observed between HFFFs infected with positive and negative HCMV clones (Figure 6B). Low HLA-E expression in the infected HFFFs is most likely due to a mutation at position 2 (Met to Val) in the canonical sequence (VMAPRTLIL) of UL40 expressed in all the TB40/E clones, as described previously (77). This result implies that the difference observed in the DT9 staining is, most likely, due to higher HLA-C expression and not HLA-E expression. In addition, assembled HLA-C and FHC of HLA-C surface levels on HFFFs infected with other viral strains, such as Merlin and AD169, were downregulated comparable to the surface levels found with the negative clones (Figure S6 in Supplementary Material). Together, these results indicate that the positive clones are less effective in downregulating assembled HLA-C and, in particular, FHC of HLA-C in HFFFs, compared to the negative clones and other HCMV strains. However, both sets of clones downregulated other HLA molecules to similar levels.

#### More Total HLA-C Protein Is Expressed on HFFFs Infected with Selected Clones of HCMV

After having found that HLA-C and, in particular, FHC of HLA-C cell-surface levels were higher on positive clone-infected HFFFs, we investigated whether differential expression of total HLA-C protein levels could be tracked by western blot. HFFFs infected with three positive clones had higher amounts of total HLA-C compared to the negative clones, although the levels in general were lower than for the uninfected and UV B6 clone samples. As expected, HFFFs stimulated with UV B6 clone contained high amounts of HLA-C protein (Figure 6C). Additionally, we tested HFFFs infected with a further 12 clones (6 positive and 6 negative, Figure S5B in Supplementary Material). From the 18 clones tested in total, 7 positive clones had high amounts of total HLA-C, while 7 negative clones had lower amounts.

In conclusion, positive TB40/E clones are defective in downregulating HLA-C and, in particular, FHC of HLA-C in HFFF cells. Higher amounts of total HLA-C protein were found in HFFFs infected with the positive TB40/E clone compared to infection with the negative TB40/E clone. This may reflect a deficiency in degradation/turnover of HLA-C by positive TB40/E clones.

### Pan HLA Class I Antibodies Block the KIR2DS1–Ligand Interaction

**Figure 7:**
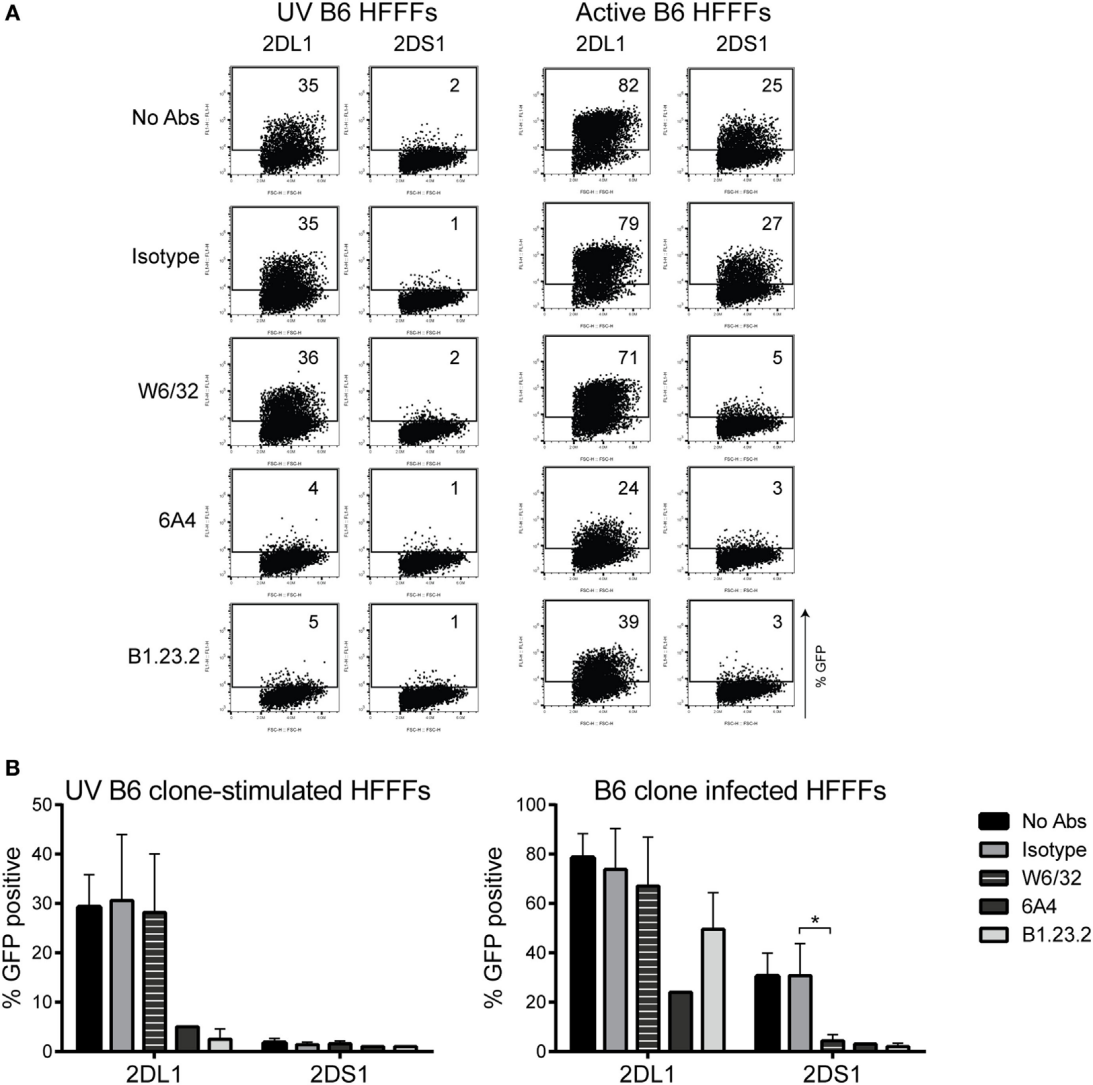
**Antibody-blocking experiment of reporter cells and HFFFs stimulated with UV B6 clone or infected with the B6 clone**. W6/32, 6A4, B1.23.2, and isotype antibodies were added to 72 h UV-stimulated or infected HFFFs, before an overnight coculture with KIR2DS1 and KIR2DL1 reporter cells was performed. GFP expression was measured by flow cytometry. **(A)** The dot plots are data from a representative experiment. **(B)** Collection of up to five independent blocking experiments is depicted in bar graphs as the mean ± SD of individual samples with *p < 0.05 calculated by non-parametric one-way ANOVA using the Kruskal–Wallis test and Dunn’s multiple comparisons test.

Antibody-blocking experiments were performed using various anti-HLA class I antibodies to obtain a better understanding of the KIR2DS1 interaction. Previously, Stewart and colleagues tested a large panel of pan HLA class I antibodies in a blocking experiment to analyze the interaction of KIR2DL1 and KIR2DS1 tetramers with 221-HLA-C transfectants (27). They concluded that W6/32 and HC10 antibodies were not able to block both KIR2DL1 and KIR2DS1 tetramer interactions with C2-HLA-C. However, other pan HLA class I antibodies, such as 6A4 and B1.23.2, did block (27). To confirm the previous findings, the interaction of KIR2DL1 reporter cells with UV B6-stimulated HFFFs, which highly expressed all HLA class I molecules, was tested. As expected, W6/32 antibody did not block the KIR2DL1 interaction with the HFFFs, but the 6A4 and B1.23.2 antibodies did (Figures 7A,B, left panels). Next, the HFFFs were infected with the positive B6 clone and the antibody blocking experiment was repeated. W6/32, 6A4, and B1.23.2 antibodies blocked the KIR2DS1 reporter cell interaction with B6 clone-infected HFFFs. Notably, the W6/32 antibody did not block and the 6A4 and B1.23.2 antibodies only partially blocked the KIR2DL1 reporter cell interaction (Figures 7A,B, right panels). Furthermore, DT9, L31, and HC10 antibodies were also not able to block the KIR2DS1–ligand interaction and the anti-β_2_M only partially (data not shown). Together, these data are consistent with a differential mode of recognition of C2-HLA-C by KIR2DL1 and KIR2DS1.

### HFFFs with a β_2_M KO Infected with Positive HCMV Clones Do Not Induce the KIR2DS1 Ligand

**Figure 8:**
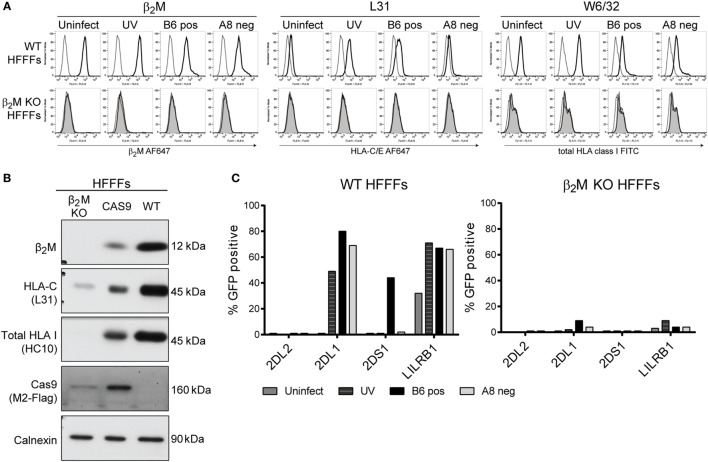
**β_2_M KO HFFFs do not activate KIR2DS1 reporter cells**. **(A)** To control the success of the β_2_M knockout, untreated (WT) HFFFs (black line) and β_2_M KO HFFFs (shaded gray line) were stained with anti-β_2_M, W6/32, and L31 antibodies. Before staining, the cells were stimulated with UV clone (UV) or infected for 72 h with the positive B6 clone (B6 pos) or the negative A8 clone (A8 neg). Unstained cells were included (dotted line). **(B)** Total lysate of β_2_M KO HFFFs, HFFFs containing CAS9 without sgRNA (CAS9) and WT HFFFs were loaded onto a reduced 10% SDS-PAGE gel. The membrane was blotted with the anti-β_2_M, L31, HC10, anti-M2 Flag, and anti-calnexin (loading control) antibodies. **(C)** β_2_M KO HFFFs and WT HFFFs were stimulated with UV B6 clone (UV) and infected with the positive B6 clone (B6 pos) or negative A8 clone (A8 neg) for 72 h. An overnight coculture was performed with KIR2DL2 (negative control), KIR2DL1, KIR2DS1, and LILRB1 reporter cells. GFP expression was measured by flow cytometry. This experiment was performed twice.

To confirm whether the KIR2DS1 ligand is a HLA class I molecule and specifically HLA-C, the β_2_M gene was knocked out of HFFFs. The HLA class I complex cannot be formed without β_2_M and therefore, HLA class I molecules, but also FHC of HLA class I, will not be transported efficiently to the cell surface (78). The *β_2_M* gene was knocked out by using the CRISPR/CAS9 genome editing tool (79). After selection and single-cell sorting, β_2_M KO HFFFs were checked for β_2_M, total HLA class I (W6/32), and FHC of HLA-C (L31) surface expression by flow cytometry and total protein expression by western blot. β_2_M KO HFFFs did not express surface β_2_M, total HLA class I, and FHC of HLA-C. B6 and A8 clone infection did not alter these expression levels (Figure 8A). Total β_2_M, total HLA class I (detected with HC10), and most HLA-C protein were also absent in the β_2_M KO HFFFs, compared to the untreated HFFFs (WT) and HFFFs containing CAS9 without the sgRNA (CAS9), indicating that the β_2_M KO was successful (Figure 8B).

Subsequently, β_2_M KO HFFFs and WT HFFFs, either stimulated with UV B6 clone or infected with a positive and negative clone, were cocultured with the KIR2DS1, -L1, -L2, and LILRB1 reporter cells. The KIR2DS1 reporter cell was not activated after encountering β_2_M KO HFFFs infected with the positive B6 clone, while, in the same experiment, 44% of KIR2DS1 reporter cells were GFP positive after coculture with B6 clone-infected WT HFFFs (Figure 8C). KIR2DL1 and LILRB1 reporter cells were not significantly triggered by β_2_M KO HFFFs in all the conditions (Figure 8C).

This result is consistent with KIR2DS1 recognizing an HLA class I molecule, including HLA-C. Together with the cell-surface staining and the antibody-blocking experiment, the data suggest that modulation of C2-HLA-C by HCMV induces a potent KIR2DS1-mediated NK cell activation.

## Discussion

We found that KIR2DS1 recognizes a ligand on HFFFs infected with the TB40/E strain of HCMV. This wild type strain consists of at least two sets of virus clones: one set that, after HFFF infection, activates KIR2DS1-expressing cells (positive clones) and one that does not (negative clones). This specific KIR2DS1 recognition was only present when the HFFFs were infected with fully functioning viruses, indicating that the virus plays a direct role in expressing the KIR2DS1 ligand. In addition, KIR2DS1 single-positive (2DS1sp) primary NK cells degranulated after engaging with positive clone-infected HFFFs. Together, this indicates that KIR2DS1 reporter cell activation correlates with physiological KIR2DS1 binding to its ligand.

### The Reporter System Is a Valid Way of Examining KIR Specificity

Using the reporter system, we confirmed that a single amino acid substitution K70T in KIR2DS1 altered the recognition from no binding to binding the same cognate ligands as KIR2DL1 (Figure 1C). This confirms the important role of residue 70 in the binding avidity to HLA-C by KIR2DL1/2DS1. These findings together with the differential recognition of HLA-C alleles by LILRB1, KIR2DL1, and -2DL2 reporter cells (Figure 2C) indicate that the reporter system is sensitive to subtle differences. One possible explanation of KIR2DL1 and -L2 reporter cells responding minimally to DFs (Figure 3B) is that there is binding of KIR to its ligand, but this binding is not strong enough to trigger a signaling cascade to activate the reporter cell. The signaling cascade will only be triggered if there is a true interaction. The degree of receptor/ligand clustering might also influence the down-stream signaling, as shown by Oszmiana et al. (80). Therefore, the reporter cells may be a more physiological system compared to Fc proteins or tetramers.

### KIR2DS1 Interacts with HLA-C but Not in the Same Way as KIR2DL1

There are several examples of “paired” immunoreceptors consisting of almost identical external moieties with positive and negative signaling tails, respectively (13). It is believed, but by no means proven, that this situation is driven by host–pathogen interaction. Our data are broadly consistent with this proposal. Our data fit with KIR2DL1, the inhibitory receptor, interacting with C2-HLA-C for recognition of a self-ligand in order to promote education/licensing of NK cells and subsequent loss of inhibition when the ligand is missing. By contrast, the role of aKIR has been unclear. Some groups reported weak binding, particularly for KIR2DS1, but these effects are inconsistent. In general, aKIR appear to interact with HLA molecules weakly except in certain circumstances (27, 41, 42). We found that KIR2DS1 reporter cells were not activated after engaging conventional HLA class I molecules (Figures 2 and 3), as shown previously by others (10, 11, 17, 24–35). There is a possibility that the target cells used in previous KIR2DS1 studies did not only express conventional HLA-C molecules. These cells were EBV positive (721.221 cells, BLCLs, C1R cells), and/or tumor-derived cells, such as leukemia blasts and lymphomas. In addition, other studies included primary cells such as DCs (33), T cells (30, 33), B cells (27, 29), and MRC-5 fibroblasts (supplementary data of Stewart et al.) KIR2DS1 did not interact with or bind these primary cells, unless they were stimulated: in the case of DCs, stimulated with LPS and T cells, stimulated with PHA to form T cell blasts. Furthermore, Crespo et al. found that HCMV-infected JEG-3 cells and fetal extravillous trophoblasts (EVT) did not induce degranulation and cytokine production of dNK cells. They only found a cytotoxic response when dNK cells were exposed to HCMV-infected DSC specifically, indicating differential recognition of dNK cells to HCMV-infected cells (47). One explanation could be that KIR2DS1-mediated NK cell activation could only occur through an unknown synergistic engagement of other activating receptors, as proposed by Bryceson et al. (81). Additionally, perhaps a high level of C2-HLA-C is needed for a potent KIR2DS1-mediated NK cell activation, which is the case for these target cells. Primary cells might express too low levels of C2-HLA-C and in combination with the weak binding of KIR2DS1 to C2-HLA-C might result in the absence of NK cell activation. Crespo et al. also found reduced levels of HLA-C on HCMV-infected DSC leading the authors to speculate that an unknown activating ligand for KIR2DS1 is upregulated by HCMV infection which is recognized by dNK cells (47). Alternatively, our findings indicate that KIR2DS1 might recognize a modified form of C2-HLA-C, which is induced by selected TB40/E clones in HFFFs. Compared to HCMV from other sources, positive TB40/E clones were less effective in controlling FHC of HLA-C and, to a certain extent, assembled HLA-C (Figure 6). W6/32, 6A4, and B1.23.2 antibodies were able to block the KIR2DS1–ligand interaction on positive clone-infected HFFFs, while these antibodies could not block, or only partially block, the KIR2DL1 interaction (Figure 7). β_2_M KO experiments confirmed that KIR2DS1 binds a HLA class I molecule, most likely HLA-C, on these infected cells (Figure 8). It is unlikely that KIR2DS1 was binding directly to an HCMV-encoded protein, since KIR2DS1 reporter cells were not activated in coculture with β_2_M KO HFFFs infected with the positive clone. The most parsimonious interpretation is that the virus influences the balance of recognition directly of HLA-C by the KIR2DL1/S1 pair. At this stage, however, other possibilities cannot be ruled out, such as a combination of HLA class I with another protein.

### What Is the Difference Between HLA-C Recognized by KIR2DS1 and by KIR2DL1?

**Figure 9:**
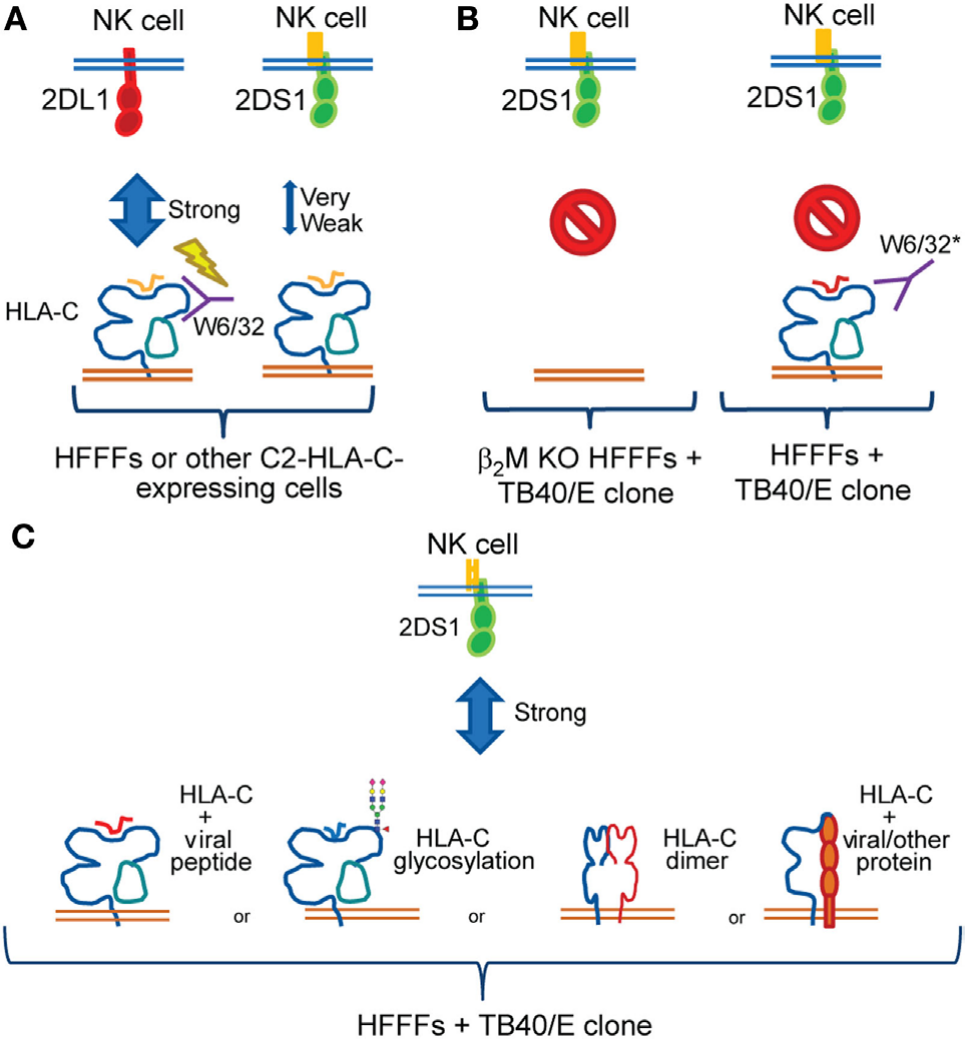
**Summary of the findings and working models**. **(A)** Both KIR2DL1 (red) and -2DS1 (green) bind conventional group C2 human leukocyte antigen (HLA)-C; however, KIR2DS1 binds weakly. Our data were consistent with previous studies demonstrating that the W6/32 antibody does not block the KIR2DL1–HLA-C interaction. The lightning bolt indicates KIR2DL1 triggering even after adding W6/32 to the coculture. **(B)** Strong KIR2DS1 activation was observed after coculture with HFFFs-infected selected TB40/E clones. After infecting β_2_M KO HFFFs with selected TB40/E clones, KIR2DS1 interaction was diminished (left). In addition, W6/32 blocked the KIR2DS1–ligand interaction after coculture with HFFFs-infected selected TB40/E clones (right). *Other pan class I antibodies, such as 6A4 and B1.23.2, blocked both KIR2DL1 and KIR2DS1 interaction. The red symbol indicates no binding of KIR2DS1 after coculture in the indicated setting. **(C)** Remaining FHC of HLA-C and, to certain extent, assembled HLA-C surface expression was found on infected HFFFs. Together with the other findings, we hypothesize that a modification of C2-HLA-C is induced by HCMV, which influences KIR2DS1 recognition. This modification could relate to the following: presentation of HCMV-derived peptides; alteration of glycosylation patterns on the HLA-C molecule; formation of HLA-C homodimers; or heterodimers association with another protein.

Since all the data indicate that HLA-C forms at least part of the KIR2DS1 ligand the question that arises is how does HLA-C differ upon viral infection such that KIR2DS1 is brought into play? Figure 9 suggests various models which may be tested, namely:

1. *Bound peptide*: there have been reports of KIRs recognizing certain peptide motifs presented by HLA class I molecules. Stewart and colleagues have demonstrated that amino acids at position 7 and 8 of the peptide play a role in KIR2DS1 binding. KIR2DS1 has similar peptide selectivity to KIR2DL1 (27). Work from Khakoo’s group has shown that peptides with certain motifs have either strong inhibitory, low inhibitory, or antagonistic effects on KIR2DL2 and -2DL3^+^ NK cells (82). These data suggest that NK cells are able to sense alterations of target cells, through selective peptide recognition.
2. *Modified glycosylation*: KIR3DL1 binding to HLA-B57:01 is dependent on the N-glycan on HLA-B57:01. Removing the N-glycan resulted in reduced inhibition, thus increasing degranulation of KIR3DL1^+^ NK cell clones (38). The N-glycosylation site on HLA class I is highly conserved and the glycan structures on HLA-C are relatively uniform between HLA-C allotypes (83). Perhaps viral infections could alter these glycosylation patterns and break the uniformity, resulting in the recognition of KIR2DS1. HIV and HCV infection has been shown to alter glycosylation in host cells, due to ER stress (84, 85). This might change the glycosylation pattern of HLA class I.
3. *Formation of HLA-C homodimers or heterodimers with other HLA class I molecules*: KIR3DL2 binds FHC forms of HLA-B27, including HLA-B27 dimers (86). LILRB1 interacts with different HLA class I molecules, which are able to dimerize via a cysteine residue in the cytoplasmic tail (49). Recently, it is described that the ligand for KIR3DS1 is FHC of HLA-F (41, 42). These data are evidence of the capability of Ig-like receptors to bind FHC HLA molecules or HLA homodimers.
4. *Modification by formation of HLA-C heterodimers with a virus-derived protein*: in mice, activating receptor Ly49P binding to an MHC-CMV protein heterodimer has been reported. Ly49P binds CMV-infected cells expressing a complex of the m04 CMV protein together with the MHC class I molecule H-2D^k^ (87). Very recently, Serena et al. proposed that HIV-1 Env protein associates with FHC of HLA-C and that HIV-1 specifically upregulates FHC of HLA-C at the cell surface of infected cells (88). Their work has some parallels with our findings. Our preliminary experiments, however, did not show any evidence of HLA-C forming homo- or heterodimers (data not shown).

Based on these observations together with our findings, we argue that KIR2DS1 most likely binds C2-HLA-C either through recognizing HCMV-derived peptide or changes in glycosylation patterns. This will be the focus of future experiments.

### Have Activating KIRs Evolved to Recognize Infected Cells?

Studies investigating the evolution of KIRs and other paired receptors have proposed that the activating members may be evolving more rapidly than the inhibitory members through selection imposed by pathogens (15, 16). The positively charged lysine at position 70 in KIR2DS1 is critical for the diminished binding to C2-HLA-C compared to KIR2DL1. This amino acid is conserved in all KIR2DS1 allotypes (with the exception of KIR2DS1*001, which has a charged arginine) (11). Conversely, the lysine at position 70 in KIR2DS1 could be crucial for binding modified HLA-C induced by pathogens. KIR2DL1 might have evolved to recognize HLA-C on healthy cells (induced-self), while KIR2DS1 might recognize slight structural changes on HLA-C induced by pathogens (altered-self). KIR2DS1 may still bind conventional HLA-C weakly to secure tolerance, yet recognition by KIR2DS1 of a modified form of HLA-C could overcome this tolerance. Our findings favor this hypothesis.

### Why Were KIR2DL1 Reporter Cells More Activated after Encountering TB40/E-Infected HFFFs?

The GFP expression of KIR2DL1 reporter cells, after encountering TB40/E-infected HFFFs, was even higher than in coculture with UV TB40/E-stimulated HFFFs, which contain high expression levels of HLA-C (Figure 4A). HCMV downregulates HLA class I molecules, thus these findings were unexpected. A possible explanation is that KIR2DL1 binds the remaining HLA-C on the infected cell surface (Figure 6B). Ameres et al. reported that HCMV downregulates certain HLA-A and -B alleles more efficiently than HLA-C alleles (89, 90). Another explanation could be that KIR2DL1 recognizes an alternative ligand, comparable to how both inhibitory Ly49I and activating Ly49H-binding m157 (43, 91).

### Differences in HCMV Isolates

Natural killer cell responses appear to differ when encountering cells infected with various HCMV strains and even clones within strains. Chen et al. also demonstrated that the ability of NK cells to control virus spread through LILRB1 was variable between HCMV viral strains, depending on the amino acid sequence within UL18 (92). Thus, the variable effect between HCMV strains on NK cell activity and vice versa should be taken into account when setting up experiments and interpreting published data. This will also count for cytotoxic T cells and other immune cell responses.

TB40/E-derived positive clones were the only viruses that, upon infecting HFFFs, expressed the KIR2DS1 ligand. Together, these findings imply that after infection the positive clones are less successful in downregulating the KIR2DS1 ligand than TB40/E-derived negative clones or other HCMV strains. As a result, KIR2DS1 reporter cells and 2DS1sp NK cells are specifically detecting the ligand on positive clone-infected HFFFs. This suggests that the other HCMV strains are capable of downregulating the KIR2DS1 ligand as an immune evasion strategy to NK cells. Our findings could explain why this interaction has not been detected in previous studies. Identification of the differences between the positive and negative clones by whole virus genome sequencing should help to resolve this issue.

## Conclusion

Our findings indicate that activating KIRs do not bind the same conventional HLA molecules as their inhibitory counterparts. They suggest that pathogenic infections are required for strong activating KIR binding, discriminating between healthy and unhealthy cells. To our knowledge, this is the first time that the role of HCMV on activating KIR recognition has been conclusively shown. Future work will provide new insights into the role of NK cells in HCMV infection and transplantation. This could lead to more targeted and effective therapeutic avenues in the treatments for HCMV infection in new-born babies, immunosuppressed individuals, and patients undergoing solid organ or HSCT transplantation.

## Ethics Statement

This study was carried out in accordance with the recommendations of Addenbrookes National Health Service Hospital Trust institutional review board, Cambridgeshire 2 Research Ethics Committee (REC 97/092) with written informed consent from all subjects. All subjects gave written informed consent in accordance with the Declaration of Helsinki. The protocol was approved by the Cambridgeshire 2 Research Ethics Committee.

## Author Contributions

KP, MW, and JT conceived and designed the experiments. KP and MI performed the experiments and analyzed the data. CC designed and provided the reporter cells. KP, MW, JT, CC, MI, and AM discussed the data and commented on the manuscript. KP and JT wrote the manuscript.

## Conflict of Interest Statement

The authors declare that the research was conducted in the absence of any commercial or financial relationships that could be construed as a potential conflict of interest.
