# Supplementary material for: Modulation of Human Leukocyte Antigen-C by Human Cytomegalovirus Stimulates KIR2DS1 Recognition by Natural Killer Cells
Source: Front Immunol. 2017 Mar 29;8:298. doi: 10.3389/fimmu.2017.00298 (PMC5372792; doi:10.3389/fimmu.2017.00298)
Supplement: Supplementary file 1 [file Image_1.pdf]

Supplementary Material

Modulation of HLA-C by human cytomegalovirus stimulates KIR2DS1 recognition by NK cells

Kattria van der Ploeg, Chiwen Chang, Martin A. Ivarsson, Ashley Moffett, Mark R. Wills*, John Trowsdale*

*** Correspondence:** John Trowsdale: jt233@cam.ac.uk, Mark Wills: mrw1004@cam.ac.uk

1 Supplementary Figures

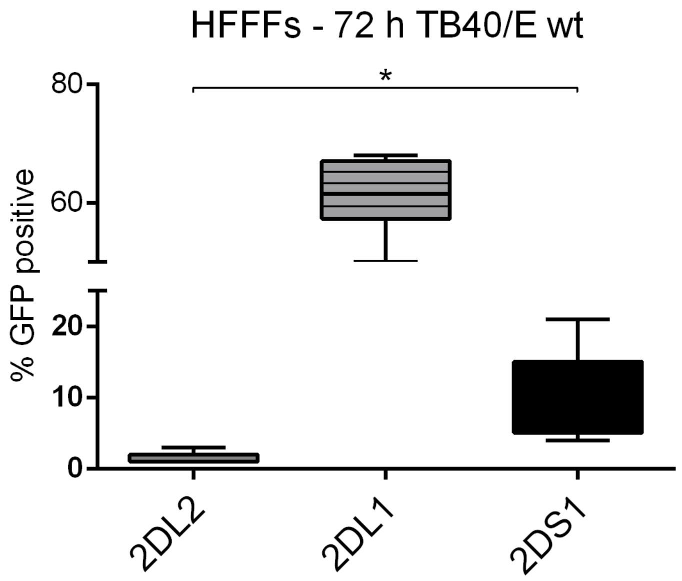

Supplement Figure 1. The KIR2DS1 reporter cell recognizes a ligand on HFFFs infected with theTB40/E wild type strain after 72 h of infection. HFFFs were infected 72 h with HCMV TB40/E wild type strain with an MOI of 10 and cocultured overnight with the reporter cells as indicated. After an overnight coculture, the GFP expression was measured using flow cytometry. A collection of 10 independent experiments is depicted. KIR2DS1 reporter cell activation is significantly different compared to the KIR2DL2 reporter cell activation (negative control) (*P<0.05, by nonparametric One-Way ANOVA using the Kruskal-Wallis test and Dunn's multiple comparisons test).

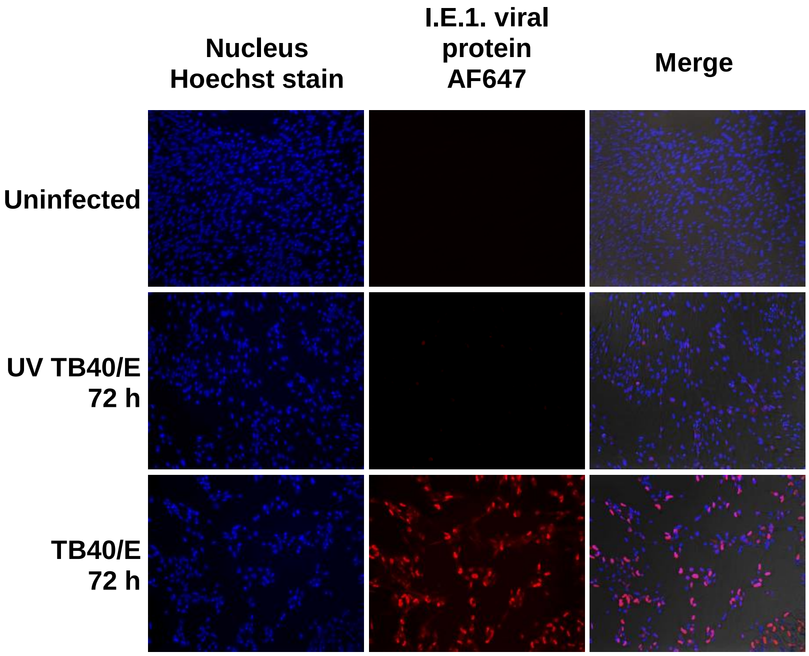

Supplement Figure 2. Immunohistochemistry staining of Immediate Early 1 (I.E.1) viral proteins. The success of TB40/E virus inactivation by UV light, thus the absence of functional viral proteins, was tested by performing immunohistochemistry staining of Immediate Early 1 (I.E.1) viral proteins on uninfected, 72 hours UV TB40/E-stimulated and TB40/E-infected HFFFs. HFFFs were treated for 72 h. 70% ethanol was added for 30 min at -20°C to permeabilize the cells. 1 µg/ml anti-HCMV I.E.1 antibody (Millipore) in PBS was added. After 1 h incubation, 2 µg/ml goat anti-mouse IgG conjugated to AlexaFluor® 594 monoclonal antibody (AF594, Life Technologies) and 1 µg/ml Hoechst (Sigma-Aldrich) in PBS were added for additional hour. Cells were visualized under UV (Hoechst) and green light (AF594) using an Axio scope A1 Epi-fluorescent Microscope (Zeiss, Cambridge, UK). I.E.1. viral proteins were absent in UV TB40/E stimulated HFFFs indicating that viral proteins have been successfully inactivated by UV light.

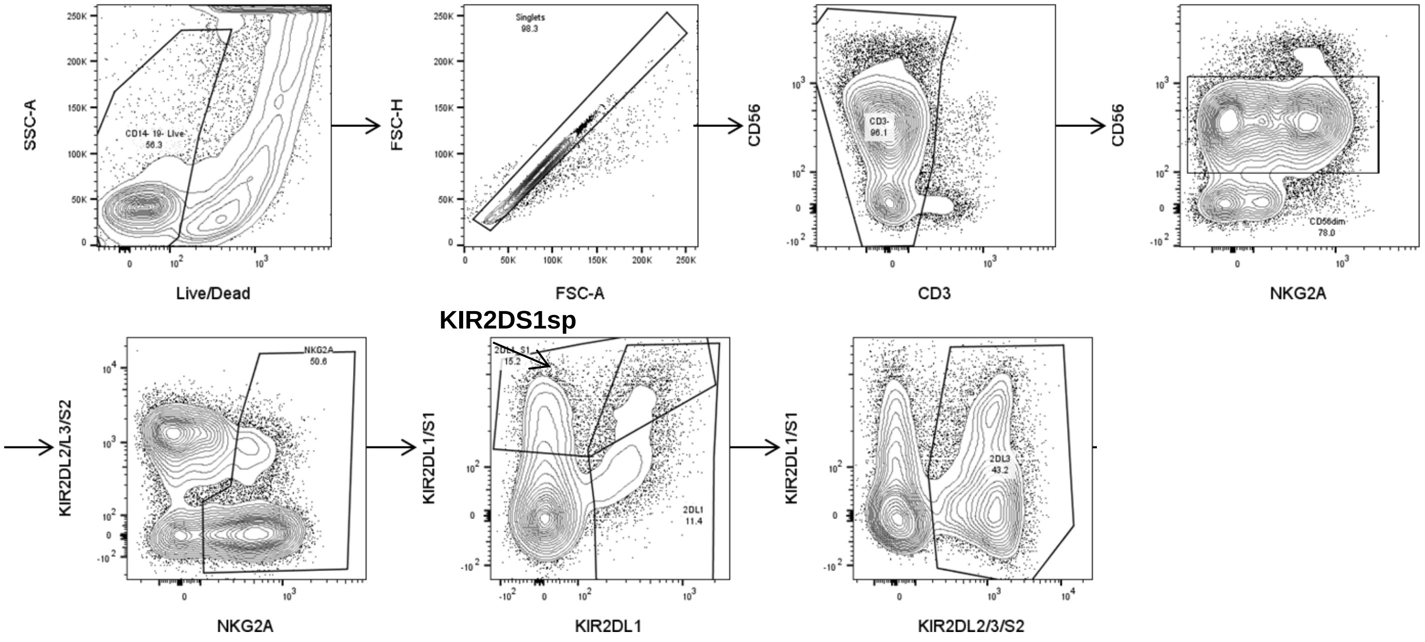

Supplement Figure 3. Gating strategy of NK cells for co-culture experiments. The NK cells were gated as described by *Fauriat et al. (2010) Blood.* In brief, dead cells, doublets and $CD3^+$ cells were excluded. The gates indicated were then used to perform Boolean-gating on NK cell subsets of interest.

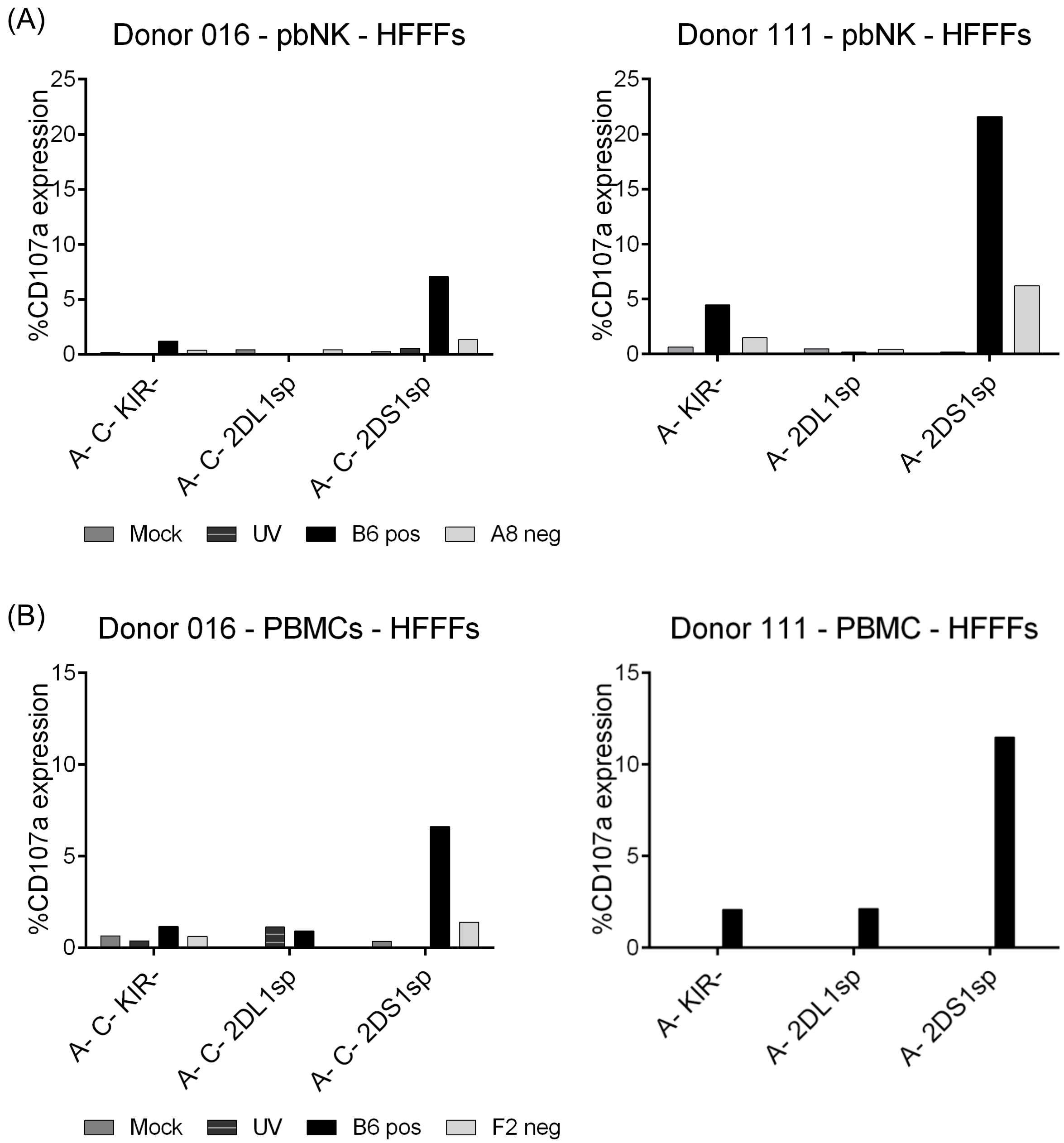

Supplement Figure 4. Primary NK cells of donor 016 and 111 degranulated after co-culture with HFFFs infected with the positive B6 TB40/E clone. (A) Unstimulated isolated NK cells or **(B)** PBMCs were cocultured for 5 h at an E:T ratio of 1:1 or 10:1, respectively, with uninfected HFFFs, UV B6 clone-stimulated HFFFs (UV), negative A8 or F2 clone- (A8 or F2 neg) and positive B6 clone-infected HFFFs (B6 pos). Note: NK cells of donor 111 cocultured with UV B6 clone-stimulated HFFFs were lost in (A). After harvesting, the cells were stained with antibodies targeting different surface markers and the degranulation marker CD107a. NK cells from the KIR negative (KIR-), KIR2DL1 single positive (2DL1sp) and KIR2DS1 single positive (2DS1sp) subsets are shown. The percentage of CD107a expression is depicted. The NK subsets are grouped on NKG2A negative (A-) and NKG2C negative (C-) NK cells.

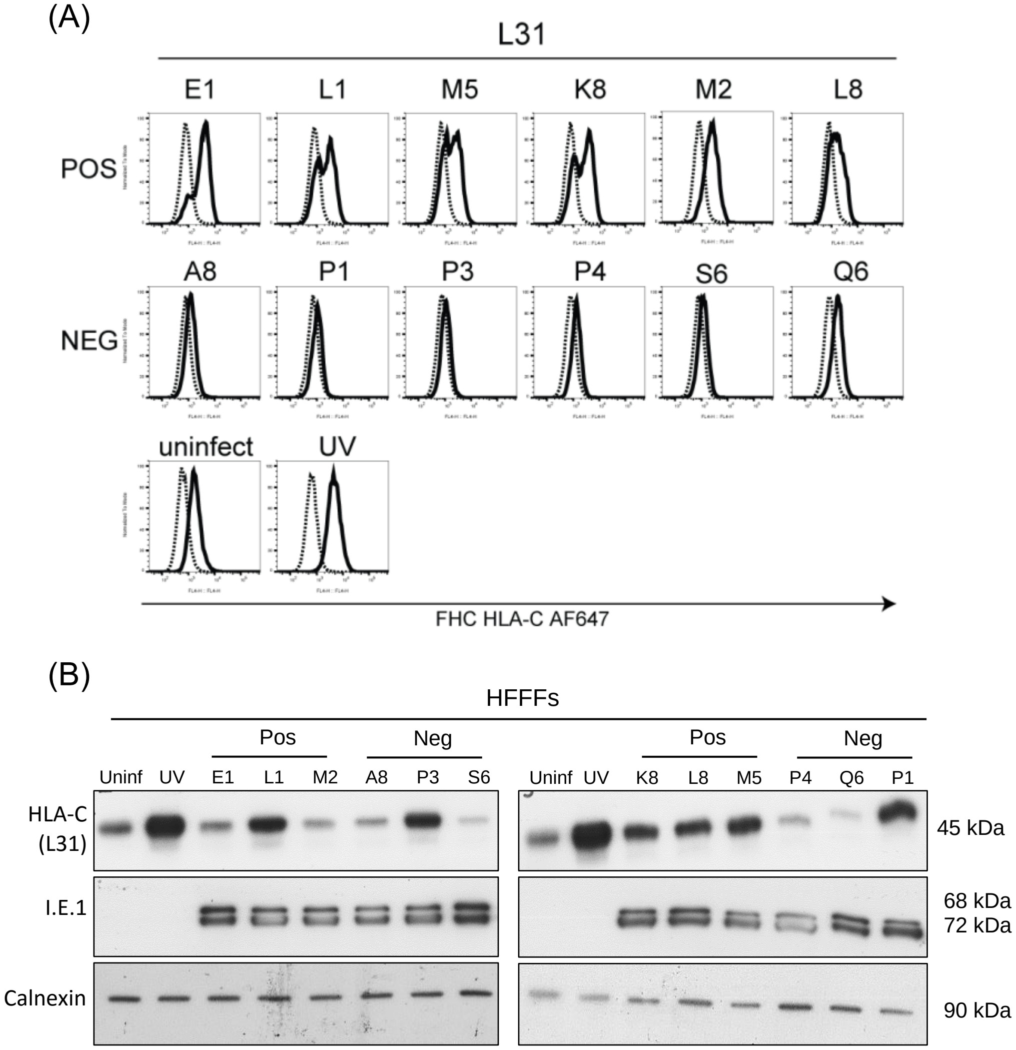

Supplementary Figure 5. **Surface FHC HLA-C and total HLA-C protein expression of HFFFs infected with 12 different TB40/E clones. (A)** Additional clones were purified from the TB40/E wild type strain. The HFFFs infected with the indicated positive or negative clones and the UV clone-stimulated HFFFs (UV) were treated for 72 h. The indicated treated HFFFs were stained subsequently with the L31 (FHC HLA-C) antibody (black line). Cells stained with the IgG1 isotype control (dotted line) were included. **(B)** Total lysate of uninfected, UV B6 clone-stimulated (UV), indicated positive clones and negative clones-infected HFFFs were loaded onto a reduced 10% SDS-PAGE gel. The membrane was blotted with the L31, anti-I.E.1 viral protein (measure of infection) and anti-Calnexin (loading control) antibodies.

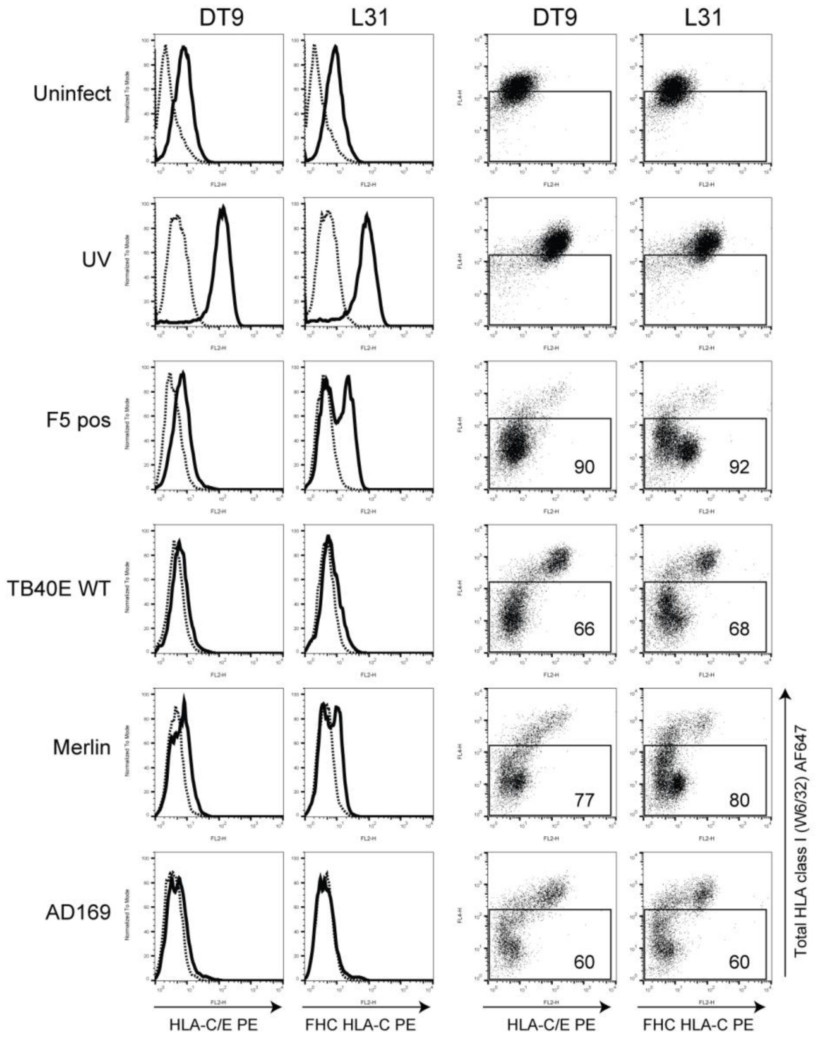

Supplementary Figure 6. Assembled and FHC of HLA-C surface expression of different HCMV strain infected HFFFs. Histograms and dot plots are presented from the same representative experiment. HFFFs were uninfected, stimulated with UV F5 clone (UV) and infected with positive F5 clone, TB40/E wildtype (TB40/E WT), Merlin and AD169 strain for 72 h. The indicated treated HFFFs were stained subsequently with W6/32 (total HLA class I), DT9 (HLA-C/-E) and L31 (FHC HLA-C) antibodies (black line). Cells stained with the appropriate isotype control (dotted line) were included. Goat anti-mouse IgG conjugated to PE was used as secondary antibody. The W6/32 monoclonal antibody is directly conjugated to AF647. The number depicted in the dot plots represents the percentage of infected cells. Data of DT9 and L31 staining of the infected HFFFs are grouped on the infected population (gates in of dot blots). This is a representative experiment of 2 independent experiments performed.
